# Modified Chaishao Liujunzi Decoction inhibits bile acid-induced gastric intestinal metaplasia: from network prediction to experimental verification

**DOI:** 10.18632/aging.205285

**Published:** 2023-12-10

**Authors:** Zheyu Sun, Yuna Liu, Haiyan Deng, Shaohua Wang, Jing Zhang, Chongyi Xing, Chunfeng Xu

**Affiliations:** 1 Changchun University of Chinese Medicine, Changchun 130021, Jilin Province, P.R. China; 2 Beijing Hospital of Integrated Traditional Chinese and Western Medicine, Beijing 100038, P.R. China

**Keywords:** gastric intestinal metaplasia, bile acid, deoxycholic acid, modified chaishao liujunzi decoction, EGFR/PI3K/AKT/mTOR

## Abstract

Modified Chaishao Liujunzi Decoction (MCLD) is a traditional Chinese medicine formula that is used mainly to improve clinical symptoms, alleviate gastric mucosal inflammation, and improve gastric mucosal lesions in patients with gastric intestinal metaplasia (GIM). GIM is considered a precancerous gastric cancer (GC) lesion (PLGC) and exploring effective intervention measures for GIM is of great importance for the prevention of GC. The purpose of this study was to reveal the potential molecular mechanism of MCLD in improving GIM induced by bile acid (BA) using network pharmacology and experimental validation. Through network pharmacology, we speculated that MCLD could act on GIM by driving the epidermal growth factor receptor (EGFR)/PI3K/AKT/mammalian target of rapamycin (mTOR) pathway. After that, we used deoxycholic acid (DCA) to treat GES-1 cells to simulate BA-induced GIM and observed the effects of MCLD treatment. The results indicate that MCLD can significantly inhibit DCA-induced cell proliferation and down-regulate the expression of pro-inflammatory cytokines and intestinal-specific markers. At the same time, MCLD also negatively regulated the expression of genes and proteins of the EGFR/PI3K/AKT/mTOR pathway. Combination with EGFR agonists and inhibitors suggested that MCLD may improve GIM by inhibiting the EGFR/PI3K/AKT/mTOR pathway, which may be related to its inhibition of DCA-induced cell proliferation through this pathway. In conclusion, MCLD may improve BA-induced GIM through the EGFR/PI3K/AKT/mTOR pathway, as predicted by network pharmacology, and is a potential Chinese medicine prescription for the treatment or reversal of GIM.

## INTRODUCTION

Gastric cancer (GC) remains a major health concern and is the fourth leading cause of cancer-related deaths worldwide, with more than one million new cases and an estimated 769,000 deaths in 2020 [1]. Intestinal-type GC follows the Correa cascade reaction, which progresses from chronic superficial gastritis, atrophic gastritis, intestinal metaplasia, dysplasia, and ultimately to GC [2, 3]. In this process, gastric intestinal metaplasia (GIM) refers to the replacement of normal gastric mucosal epithelial cells by intestinal epithelial cells. It should be noted that GIM is a recognized risk factor for intestinal-type GC and one of the most common precancerous lesions of GC (PLGC) [2, 4]. A cohort study involving 61,701 patients with GIM who were followed for 10 years showed that the annual incidence rate of GC reached 0.25% [5]. Therefore, exploring effective drugs for treating GIM to delay or reverse GIM and prevent its transition to GC is of great significance.

Hyperproliferation of gastric mucosal cells is an early molecular change in the formation of GIM. Chronic inflammation stimulates the gastric mucosa for a long time, which can induce abnormal cell proliferation, promote morphological and functional variations of cells, and then induce GIM or promote the development of GIM toward GC [6–8]. Bile reflux is an important factor that triggers or accelerates chronic inflammation [9]. Deoxycholic acid (DCA), as a hydrophobic secondary bile acid (BA), is the main component of bile reflux [10]. Exposure to DCA can promote the proliferation of gastric epithelial cells and induce GIM [11, 12]. Significantly, inhibiting this abnormal proliferation can improve DCA-induced GIM [12, 13].

Caudal-related homeobox 2 (CDX2) is a specific transcription factor expressed only in the intestine, which can regulate the expression of the downstream intestinal marker mucin 2 (MUC2) and participate in the development and differentiation of intestinal epithelial cells [14]. CDX2 does not exist in normal gastric mucosa but exhibits high ectopic expression levels in GIM tissue [15]. The study of transgenic mice confirms that CDX2 can directly regulate the transcription of intestinal specific molecules to induce GIM and intestinal-type GC [16]. Meanwhile, multiple studies have reported that DCA can induce GIM by stimulating ectopic expression of CDX2 in human gastric mucosal epithelial cells of GES-1 [14, 17, 18]. These indicate that researchers have identified the ectopic activation and expression of CDX2 as a marker of GIM.

**Figure 1:**
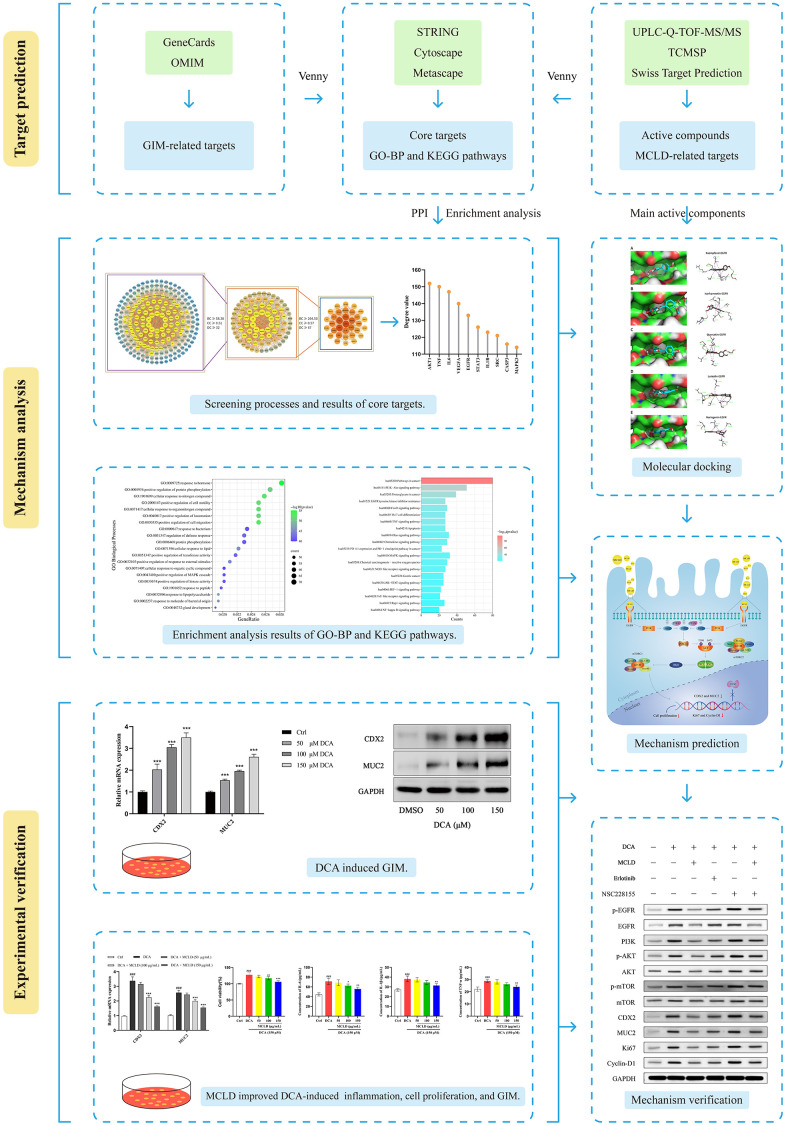
Workflow used to explore the molecular mechanism of MCLD in the treatment of GIM.

Traditional Chinese medicine (TCM) has accumulated a wealth of experience in the treatment of GIM based on the "holistic view" and the "preventing disease from exacerbating" strategy. As an alternative therapy, TCM prescriptions have been extensively studied to reveal their potential therapeutic effects on GIM [4, 19, 20]. Modified Chaishao Liujunzi Decoction (MCLD) is a TCM formula that is mainly used to improve clinical symptoms, alleviate gastric mucosal inflammation, and improve gastric mucosal lesions in patients with GIM. However, the molecular mechanism for treating GIM with MCLD is still unclear. In this study, we used network pharmacology to predict the key targets and potential molecular mechanisms of MCLD to improve GIM. At the same time, DCA was used to induce GIM by stimulating ectopic expression of CDX2 in GES-1 cells, and we further validated the potential molecular mechanism of MCLD involved in improving GIM *in vitro*. The study workflow is illustrated in Figure 1.

## RESULTS

### Chemical profiling in the water extract of MCLD identified by UPLC-Q-TOF-MS/MS

**Figure 2:**
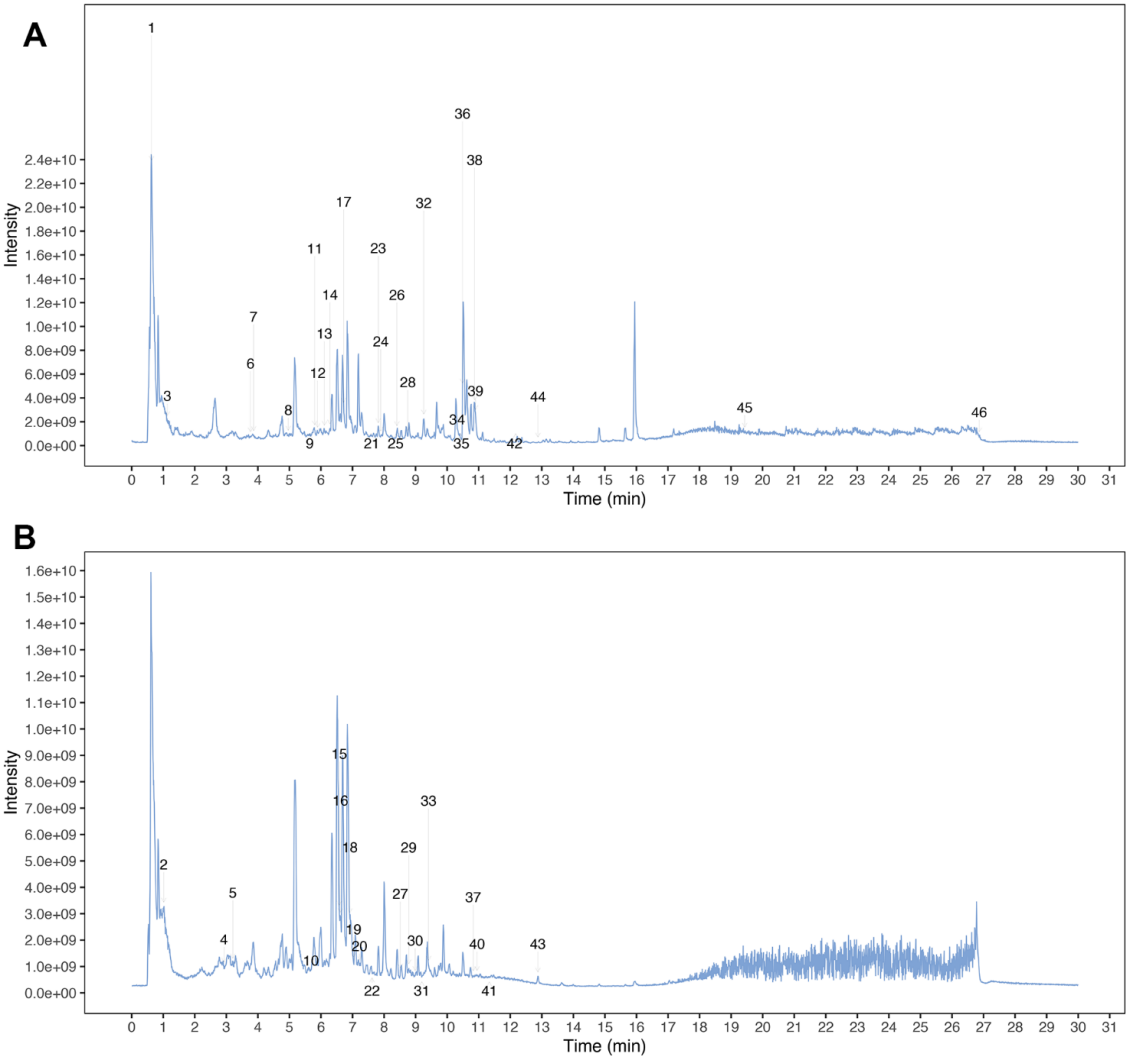
**Mass spectrum chromatograms of MCLD.** (**A**) Positive total ion chromatogram of MCLD; (**B**) Negative total ion chromatogram of MCLD.

MCLD was prepared from Radix bupleuri, Radix Paeoniae Alba, Codonopsis pilosula, Poria cocos, Atractylodes macrocephala, Pinellia ternata, tangerine peel, Coix seed, Fructus aurantii, Rhizoma cyperi, Radix notoginseng, Rhizoma zedoariae, and Herba hedyotis diffusa at a ratio of 10: 12: 15: 15: 15: 9: 10: 20: 10: 10: 3: 9: 20. Next, we obtained the total ion chromatograms (TIC) of MCLD using ultraperformance liquid chromatography coupled with quadrupole time of flight tandem mass spectrometry (UPLC-Q-TOF-MS/MS) analysis, as shown in Figure 2. By comparing retention time, precise relative molecular weight, mass charge ratio, and secondary mass spectrometry fragmentation information provided by the chromatogram with the literature, we identified the stable components of MCLD (Supplementary Table 1), which included quercetin, kaempferol, isorhamnetin, luteolin, and naringenin.

### Screening of active compounds and related target proteins

**Figure 3:**
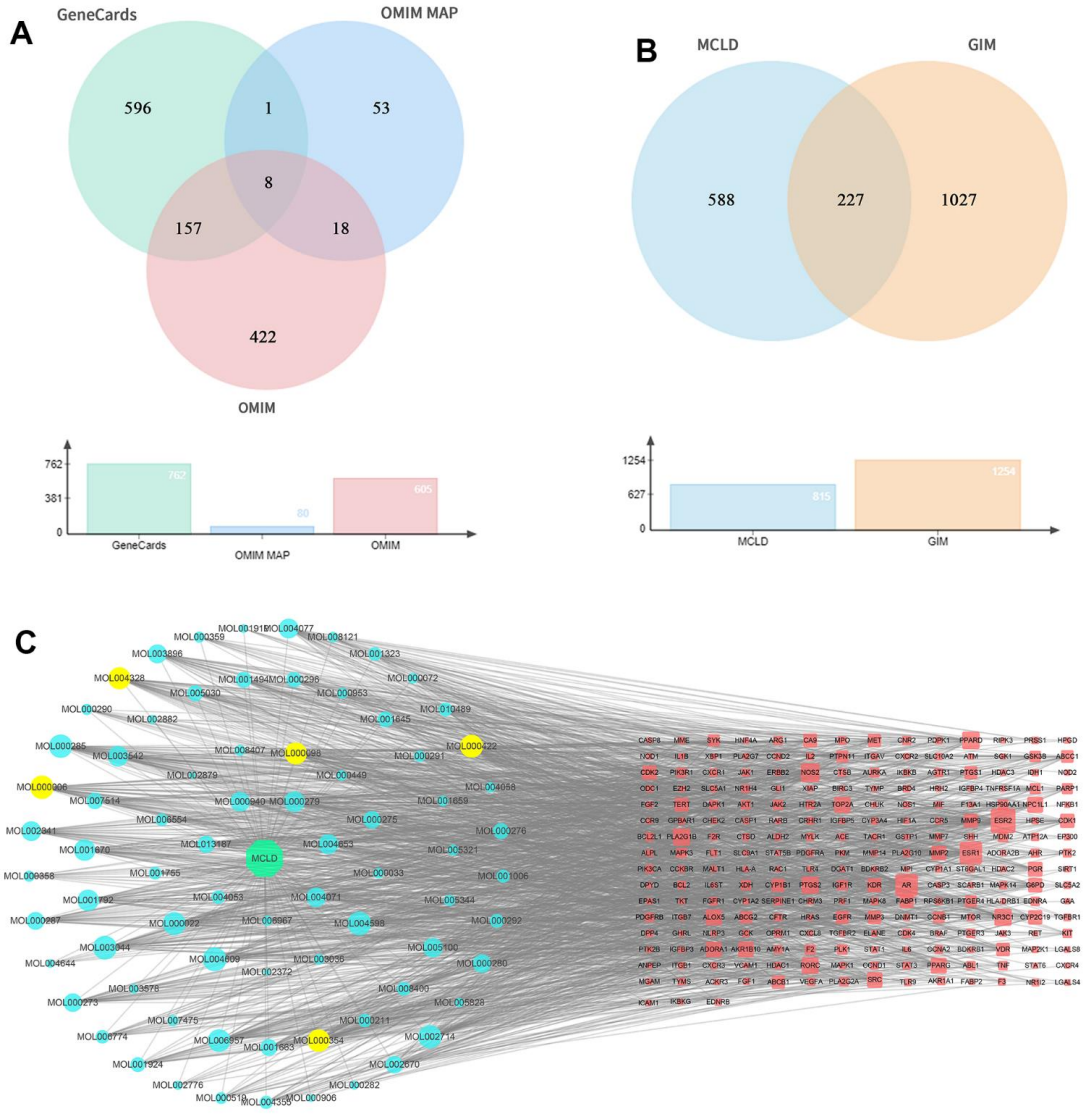
**GIM-related targets, common targets, and drug-compound-common target network.** (**A**) GIM target information is obtained through relevant databases. (**B**) Venny diagram of common targets for MCLD and GIM. (**C**) Drug-compound-common target network. The red nodes represented the targets of MCLD compounds, whereas the blue and yellow nodes represented MCLD compounds. MOL000098: Quercetin; MOL000422: Kaempferol; MOL000354: Isorhamnetin; MOL000006: Luteolin; MOL004328: Naringenin.

Oral bioavailability (OB) and drug similarity (DL) are two important parameters to evaluate the absorption, distribution, metabolism, and excretion (ADME) characteristics of drugs in the human body [21]. Based on the UPLC-Q-TOF-MS/MS identification results and database screening results, we obtained 141 MCLD-active compounds using the parameters of OB and DL (Supplementary Table 2). Using PubChem and SwissTargetPrediction databases, 815 target proteins corresponding to active compounds of MCLD were predicted (Supplementary Table 3). Furthermore, 1255 GIM-related target genes were obtained from the OMIM and GeneCards databases, as shown in Figure 3A. A total of 227 MCLD and GIM intersection target proteins were obtained using Venny v.2.1 (Figure 3B), representing the potential target proteins for drugs that act on GIM evaluated in this study. By constructing the drug-compound-common target network (Figure 3C), we obtained unique active ingredients with high degree values, including quercetin, kaempferol, isorhamnetin, luteolin, and naringenin. Therefore, we consider the above compounds as the main active ingredients of MCLD.

### Protein-protein interaction network analysis and core target screening

**Figure 4:**
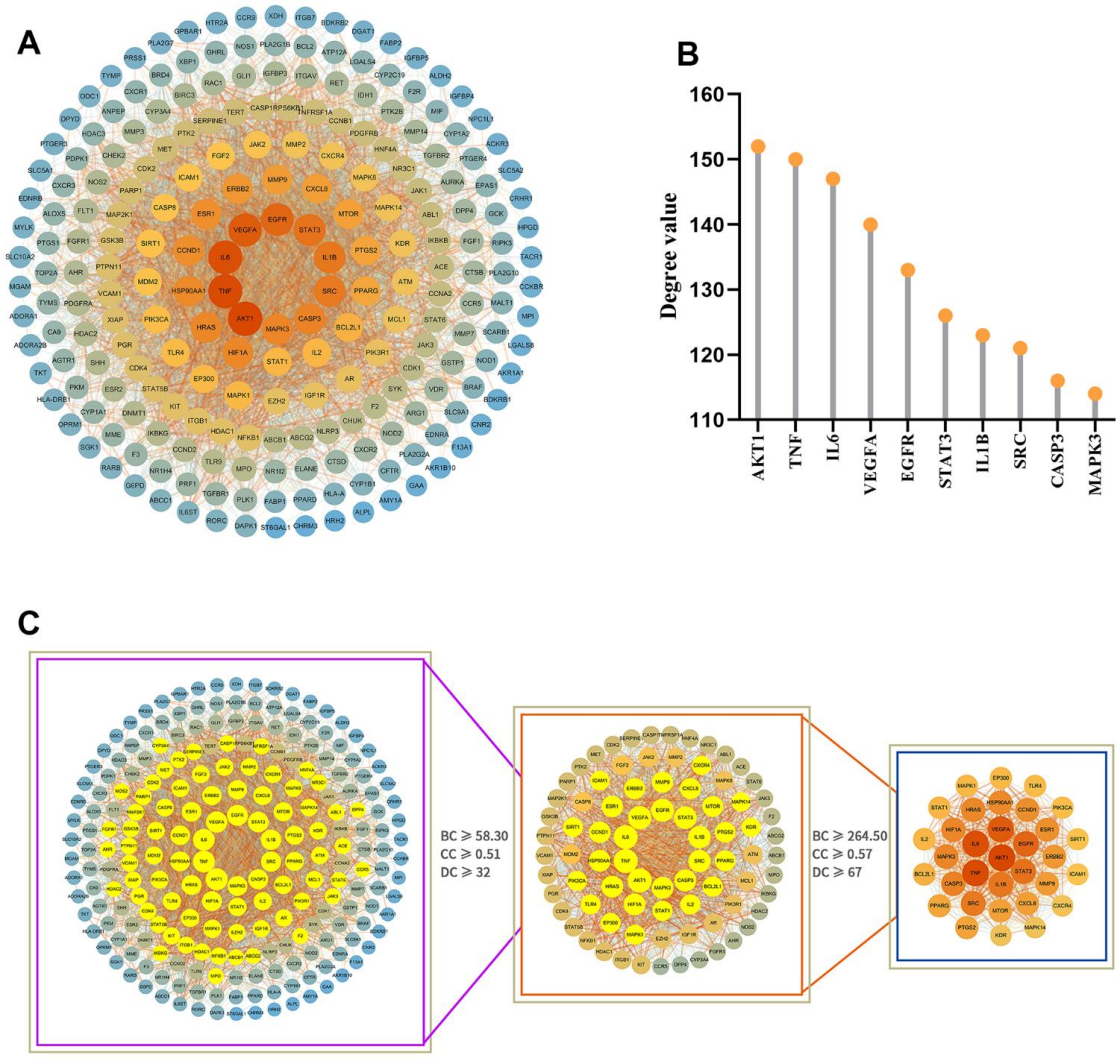
**Analysis of the PPI network of potential target proteins and selection of core targets.** (**A**) PPI network diagram of the GIM-related protein of the MCLD intervention constructed by Cytoscape software. (**B**) The top 10 core targets were selected according to degree value. (**C**) Selection of core targets based on the relevant topology analysis parameters.

To further understand the interaction between potential target proteins, we combined the STRING database and Cytoscape software to build a protein-protein interaction (PPI) network diagram (Figure 4A), where the degree value size reflected the correlation between nodes, and the size and intensity of the color of the nodes in the network were proportional to the degree value size. After two median screenings based on the relevant parameters of the topological analysis, 33 core action targets with strong interaction relationships were obtained (Figure 4C), suggesting that these may play an important biological role in the GIM intervention. According to the degree value, the top 10 core targets with the strongest correlations were AKT1, TNF, IL6, VEGFA, EGFR, STAT3, IL1B, SRC, CASP3, and MAPK3 (Figure 4B).

### Enrichment analysis using gene ontology and Kyoto encyclopedia of genes and genomes tools

**Figure 5:**
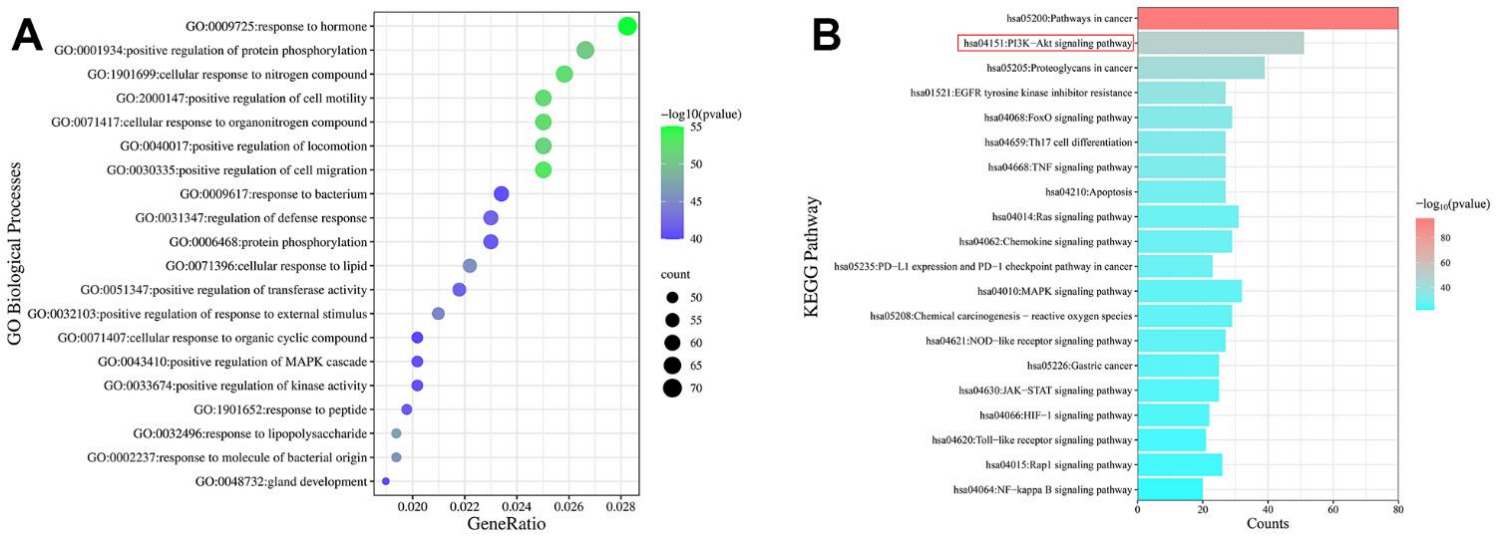
**Enrichment analysis of the GO and KEGG pathways of potential targets.** (**A**) Bubble diagram of the top 20 GO enrichment analyses identified according to biological process. The size and color of the bubbles represented the number of genes and the threshold value of the *P*-value of potential target enrichment in the biological process. (**B**) The top 20 pathways enriched by the KEGG pathway. The x-axis represents the number of targets enriched in the pathway, and the color represents the *P*-value.

To better understand the potentially complex mechanism of MCLD acting on GIM, we used Metascape and Bioinformatics online tools to conduct Gene Ontology (GO) and Kyoto Encyclopedia of Genes and Genomes (KEGG) enrichment analyses using the potential target proteins identified above and visualize the top 20 enrichment results with the highest significance according to the log *P*-value (Figure 5 and Supplementary Table 4). The results of the GO enrichment analysis suggested that 227 potential targets were enriched in a variety of biological processes related to GIM regulation, including positive regulation of cell migration, cellular response to nitrogen compounds, positive regulation of protein phosphorylation, and positive regulation of the MAPK cascade (Figure 5A) [22–24].

The enrichment analysis of the KEGG pathways showed that 137 potential targets were enriched in the top 20 pathways (Figure 5B). Interestingly, the PI3K/Akt signaling pathway, FoxO signaling pathway, and MAPK signaling pathway have been reported to be closely related to GIM, and intervention in these pathways can effectively improve GIM [4, 25, 26]. Combined with the core targets and the available literature, EGFR, and mTOR have been reported to be the upstream and downstream targets of the PI3K/Akt signaling pathway, respectively, and the EGFR/PI3K/Akt/mTOR pathway is an important pathway related to cell proliferation, growth, and differentiation. TCM prescriptions have been reported to effectively intervene in PLGC (including chronic atrophic gastritis [CAG], GIM, and dysplasia) via the PI3K/Akt/mTOR or EGFR/PI3K/Akt pathways [4, 27]. Therefore, whether MCLD acts on GIM through the EGFR/PI3K/Akt/mTOR pathway became the focus of experimental validation studies.

### Molecular docking

**Figure 6:**
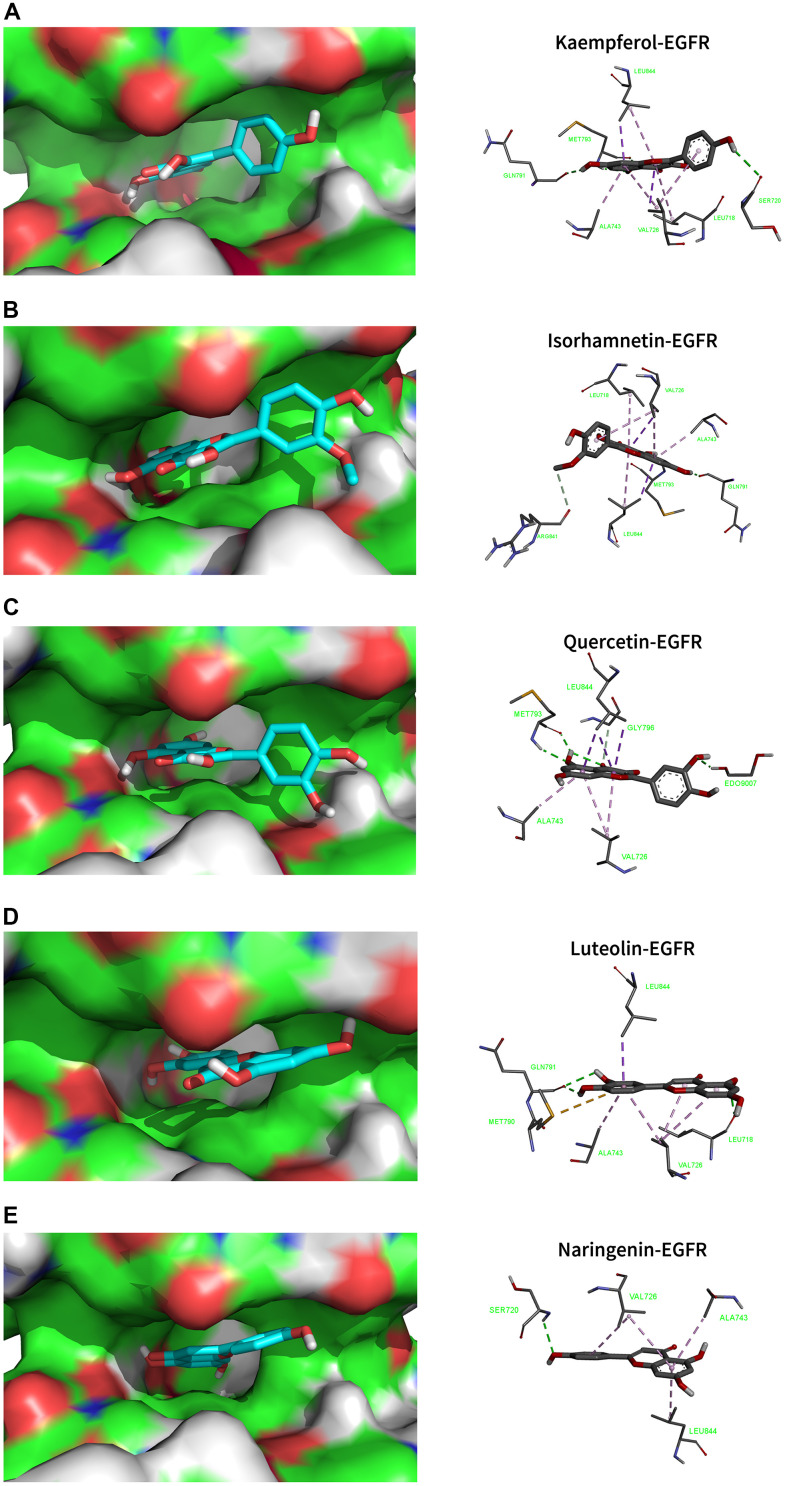
**Molecular docking models that combined the main active ingredients with the core target EGFR.** EGFR-PDB ID: 5UG9. Green dotted line: Conventional Hydrogen Bond; Light green dotted line: Carbon Hydrogen Bond; Purple dotted line: Pi Sigma; Light purple dotted line: Pi Alkyl. (**A**) Kaempferol. (**B**) Isorhamnetin. (**C**) Quercetin. (**D**) Luteolin. (**E**) Naringenin.

Using molecular docking simulations, the interaction between the active components of drugs and target proteins can be identified. In this study, we used the main active components of MCLD, quercetin, kaempferol, isorhamnetin, luteolin, and naringenin, to define the binding properties with the top ten core targets (AKT1, TNF, IL6, VEGFA, EGFR, STAT3, IL1B, SRC, CASP3, and MAPK3). In general, the lower the binding energy between the ligand and the receptor, the more stable the binding conformation [28]. Binding energy less than -4.25 kcal/mol (1 kcal ≈418,585 kJ), -5.0 kcal/mol, or -7.0 kcal/mol indicates a certain, good, or strong binding activity between the ligand and the receptor, respectively [28]. Our research results indicate that the main active components of MCLD have a good binding ability to the ten core targets mentioned above (Table 1). Among them, the binding energies of EGFR with kaempferol, isorhamnetin, quercetin, luteolin, and naringenin are -8.9, -9.0, -8.7, -9.0, and -8.8 (kcal/mol), respectively. The visualization results are shown in Figure 6, indicating that the high binding activity of the main active components of MCLD with EGFR may support the potential ability of MCLD to regulate the EGFR/PI3K/Akt/mTOR pathway predicted by network pharmacology.

**Table 1:** Binding energy of main active ingredients with the core targets (kcal/mol).

**Receptors**	**Ligands**				
	**Kaempferol**	**Isorhamnetin**	**Quercetin**	**Luteolin**	**Naringenin**
AKT1	-8.7	-8.6	-8.8	-7.4	-7.6
TNF	-7.4	-7.6	-7.4	-7.9	-8.3
IL6	-6.2	-6.4	-6.4	-7.7	-8.6
VEGFA	-5.9	-5.9	-6.3	-7.8	-6.8
EGFR	-8.9	-9.0	-8.7	-9.0	-8.8
STAT3	-5.5	-5.4	-5.5	-7.4	-6.3
IL1B	-5.1	-5.2	-5.2	-7.5	-7.2
SRC	-7.7	-8.0	-8.1	-6.5	-9.8
CASP3	-6.4	-6.7	-6.9	-5.1	-5.4
MAPK3	-9.1	-9.0	-9.2	-8.0	-9.4

### Effects of DCA and MCLD on the viability/proliferation of GES-1 cells

**Figure 7:**
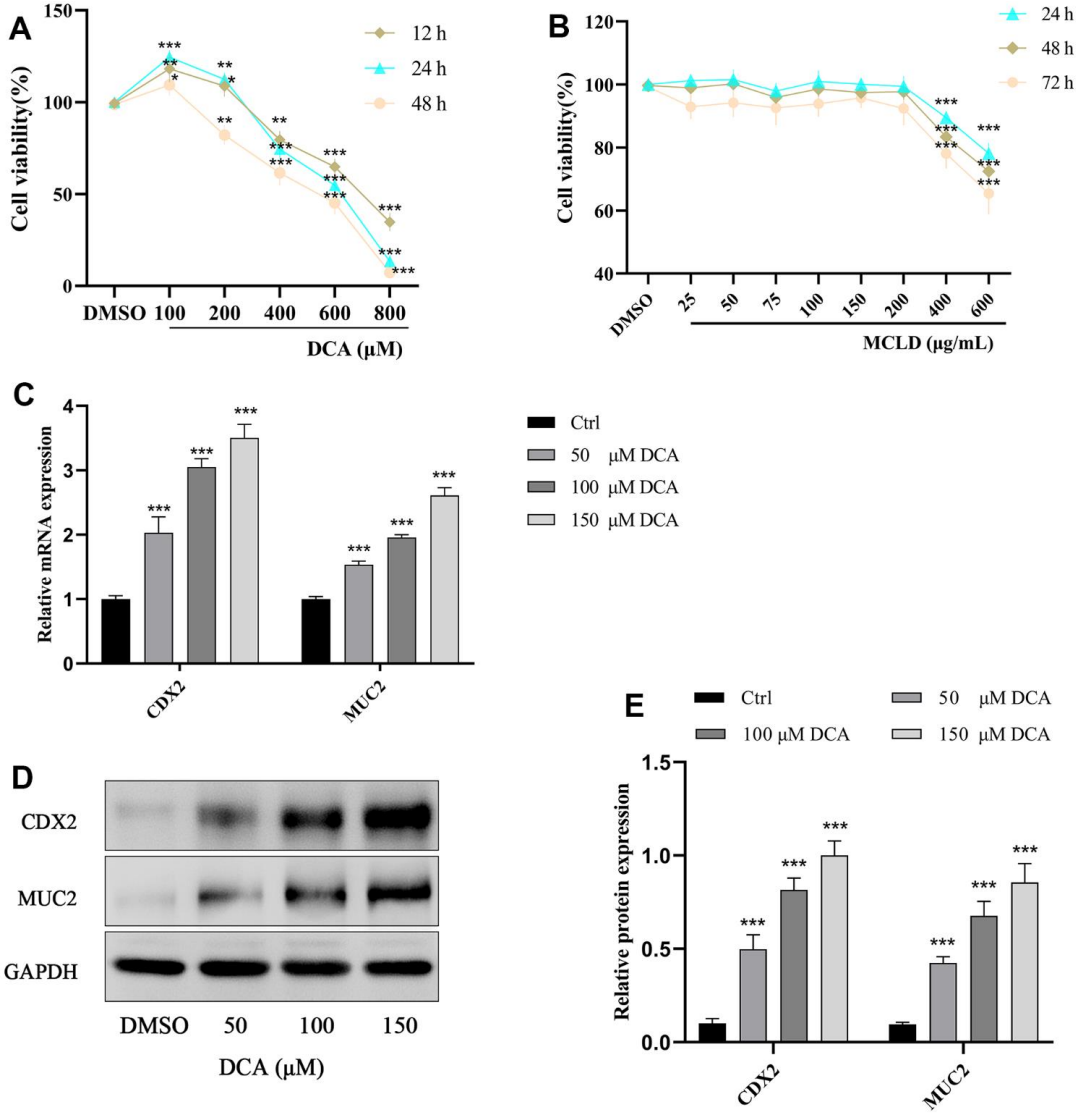
**Effects of different concentrations of DCA and MCLD on the viability of GES-1 cells at different times and DCA induced the expression of CDX2 and MUC2 mRNA and protein in GES-1 cells.** (**A**) Effects of different concentrations of DCA on the viability of GES-1 cells at different times. (**B**) Effects of different concentrations of MCLD on the viability of GES-1 cells at different times. (**C**) Effects of DCA on the expression of CDX2 and MUC2 mRNA in GES-1 cells detected by RT-qPCR. (**D**, **E**) Effects of DCA on the expression of CDX2 and MUC2 proteins in GES-1 cells detected by western blotting. Data are expressed as mean±standard deviation (SD). Compared to the control group, ^*^ *P* <0.05, ^**^P <0.01, ^***^P <0.001.

To evaluate the effects of DCA and MCLD on GES-1 human gastric mucosal epithelial cell viability, cells were treated with different doses of DCA (100–800 μM) and MCLD (25–600 μg/mL) at different times, followed by cell viability measurements using the MTT assay. Our results indicate that, compared to the blank control group, stimulation of GES-1 cells with 100 μM DCA over any period of time can enhance cell viability and promote cell proliferation. Treatment with 200 μM DCA also exhibited proliferative activity after stimulating cells for 12 or 24 h, but cell viability decreased significantly after 48 h, whereas 400 μM or above concentrations of DCA showed significant cytotoxicity at any time period (Figure 7A). Regardless of the treatment period, 200 μg/mL or lower concentrations of MCLD did not have an effect on cell viability (Figure 7B). To avoid the toxic effect of drugs on cells, low dose DCA (≤200 μM) and MCLD (≤200 μg/mL) were selected to treat GES-1 cells for 24 h in subsequent experiments.

### DCA induced the expression of CDX2 and its downstream intestinal marker MUC2 in GES-1 cells

GES-1 cells were treated with different concentrations of DCA (0, 50, 100, and 150 μM) for 24 h, and then the expression of CDX2 and its downstream intestinal marker MUC2 were detected by RT-qPCR and western blotting. As shown in Figure 7C–7E, treatment of GES-1 cells with DCA increased the expression of CDX2 and MUC2 mRNA and proteins in a dose-dependent manner, among which the 150 μM DCA treatment showed a significantly higher expression than the blank control group (*P* <0.01). These results showed that DCA could induce the expression of CDX2 and the intestinal marker MUC2 in GES-1 cells, and thus 150 μM DCA was selected for further study.

### MCLD inhibited DCA-induced hyperproliferation of GES-1 cells and levels of inflammatory cytokines

**Figure 8:**
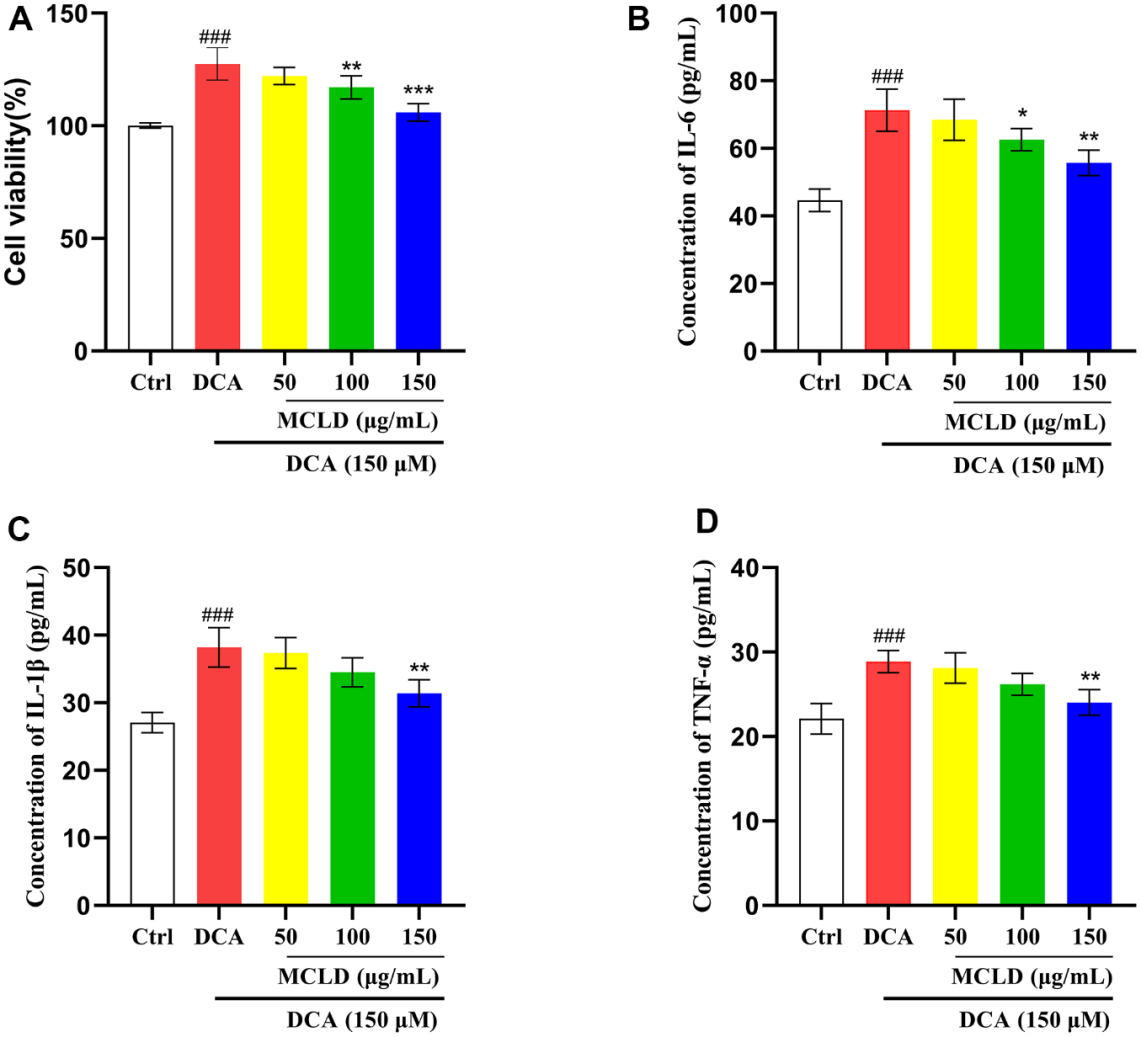
**MCLD inhibited DCA-induced GES-1 cell hyperproliferation and levels of inflammatory cytokines.** (**A**) The effect of MCLD on DCA-induced proliferation of GES-1 cells. (**B**–**D**) Effects of MCLD on the levels of inflammatory cytokines induced by DCA. Data are expressed as mean±SD (n=3). Compared to the control group, ^###^ *P* <0.001; Compared to the DCA group, ^*^ *P* <0.05, ^**^P <0.01, ^***^P <0.001.

To determine whether MCLD could improve DCA-induced GES-1 cell proliferation and levels of inflammatory cytokines, GES-1 cells were treated with 150 μM DCA for 24 h in the presence or absence of MCLD (50, 100, and 150 μg/mL), respectively. The results of MTT and ELISA showed that the proliferation activity of cells and the level of inflammatory markers (including interleukin [IL]-6, IL-1β, and tumor necrosis factor α [TNF-α]) induced by 150 μM DCA were significantly higher than those of the control group (*P*<0.01). After administering MCLD, the above indicators were inhibited, with 150 μg/mL MCLD treatment showing particularly significant (*P*<0.01) inhibition, as shown in Figure 8A–8D. These results suggest that MCLD may have a certain inhibitory effect on DCA-induced GES-1 cell proliferation and chronic inflammation.

### MCLD inhibited the target genes of the EGFR/PI3K/AKT/mTOR pathway and the expression of CDX2 and MUC2 mRNA

**Figure 9:**
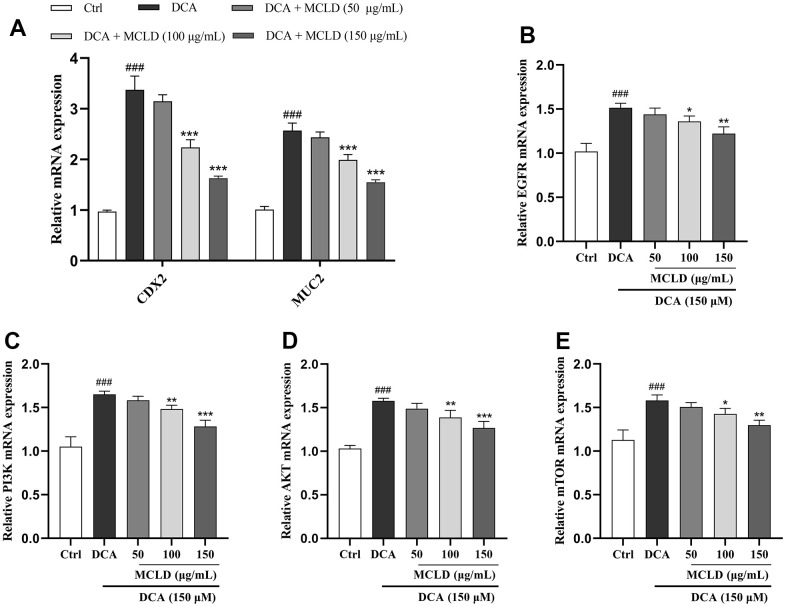
**MCLD inhibited the target genes of the EGFR/PI3K/AKT/mTOR pathway and the expression of CDX2 and MUC2 mRNA.** (**A**) Effects of MCLD on the expression of DCA-induced CDX2 and MUC2 mRNA. (**B**–**E**) Effects of MCLD on the target genes of DCA-induced EGFR/PI3K/AKT/mTOR pathway. Data are expressed as mean±SD (n=3). Compared to the control group, ^###^ *P* <0.001; Compared to the DCA group, ^*^ *P* <0.05, ^**^P <0.01, ^***^P <0.001.

Based on the results of the previous network pharmacology and molecular docking analyses, we preliminarily validated the effect of MCLD on target genes of the EGFR/PI3K pathway and the expression of CDX2 and MUC2 mRNA. The expression of these genes was detected by RT-qPCR. As shown in Figure 9, 150 μM DCA promoted the expression levels of EGFR, PI3K, AKT, mTOR, CDX2, and MUC2 mRNA, and 150 μg/mL MCLD significantly inhibited the above situation. In a subsequent experiment, 150 μg/mL MCLD was selected for further validation of the mechanism.

### MCLD inhibited DCA-induced GES-1 cell proliferation and GIM through the EGFR/PI3K/AKT/mTOR pathway

**Figure 10:**
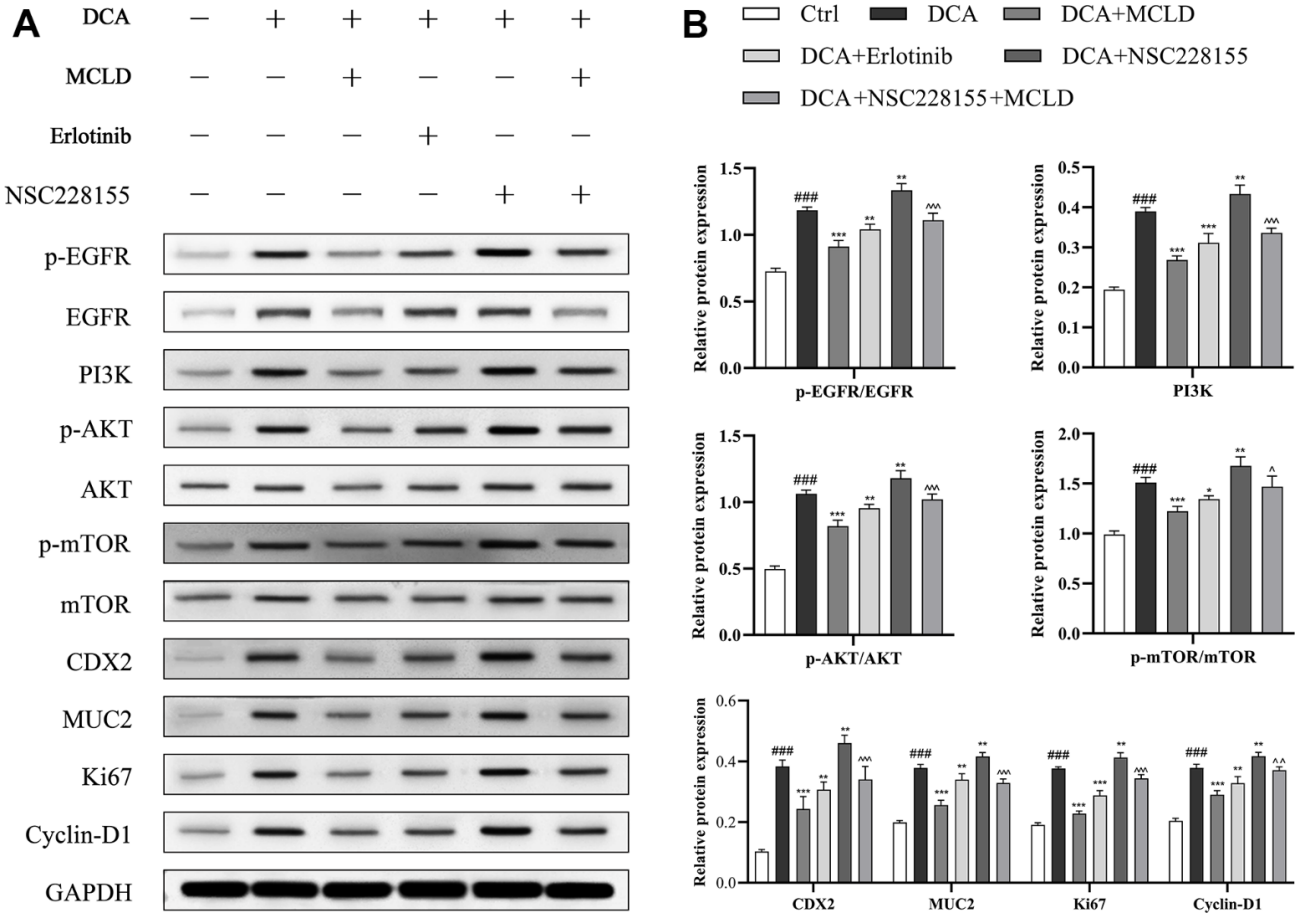
**MCLD inhibited cell proliferation and GIM through the EGFR/PI3K/AKT/mTOR pathway.** (**A**, **B**) Western blotting was used to detect the protein expression of EGFR, p-EGFR, PI3K, p-AKT, AKT, mTOR, p-mTOR, Ki67, Cyclin D1, CDX2, and MUC2 in DCA-induced GES-1 cells. Data are expressed as mean±standard deviation (SD) (n=3). Compared with the control group, ^###^ *P* <0.001; Compared with the DCA group, ^*^ *P* <0.05, ^**^P <0.01, ^***^P <0.001; Compared with the DCA+NSC228155 group, ^^^ *P* <0.05, ^^ ^^*P* <0.01, ^^ ^ ^^*P* <0.001.

To further determine whether MCLD could improve GIM by regulating the EGFR/PI3K/AKT/mTOR pathway, as predicted by our network pharmacology findings, we treated GES-1 cells with different combinations of 150 μM DCA, 150 μg/mL MCLD, 100 μM EGFR agonist (NSC228155), and 2 μM EGFR inhibitor (Erlotinib) for 24 h (Figure 10A). Western blotting analysis showed that, compared to the control group, DCA treatment significantly increased the protein expression of CDX2, MUC2, p-EGFR, PI3K, p-Akt, p-mTOR, as well as Ki67 and Cyclin D1 proteins related to cell proliferation, while MCLD and EGFR inhibitor negatively regulated the high expression of DCA-induced proteins, contrary to the result of EGFR agonist treatment. Significantly, compared to the EGFR inhibitor, MCLD exhibited a stronger inhibitory ability on the expression of the aforementioned proteins. Furthermore, compared to DCA-induced cells treated with EGFR agonist, MCLD combined with EGFR agonist significantly decreased the expression of the above proteins, as shown in Figure 10B. These data suggest that MCLD may inhibit DCA-induced cell proliferation and GIM through the EGFR/PI3K/AKT/mTOR pathway.

## DISCUSSION

GIM belongs to PLGC and is associated with an increased risk of cancer [2, 29, 30]. However, treatment options for GIM are limited, and finding effective alternatives for GIM has been a major challenge for gastroenterologists. As an alternative therapy, TCM formulas can play a potential role in treating GIM through anti-inflammatory, antibacterial, antioxidant, regulating cell proliferation or apoptosis, and maintaining immune homeostasis [19]. In this study, we combined network pharmacology and experimental validation to explore the potential molecular mechanisms of MCLD in the treatment of GIM.

Using UPLC-Q-TOF-MS/MS and network pharmacology, we predicted 141 active components and 227 potential targets that could improve GIM. The main active ingredients identified include quercetin, kaempferol, isorhamnetin, luteolin, and naringenin. The core targets included AKT1, TNF-α, IL6, VEGFA, EGFR, STAT3, and IL1B. The results of the KEGG enrichment analysis showed that MCLD can improve GIM through multiple pathways, such as the PI3K/Akt signaling pathway, the FoxO signaling pathway, and MAPK signaling. These data suggest that MCLD could act on GIM through multiple components, targets, and pathways. Combined with core targets, enrichment pathway analyses, and literature consulted, we performed a follow-up experimental verification of the involvement of the EGFR/PI3K/AKT/mTOR pathway. Of note, the results of molecular docking simulations demonstrated that the main active components of MCLD showed good binding ability with the core target EGFR. Furthermore, we found that MCLD could improve GIM by inhibiting DCA-induced cell inflammation and interfering with the EGFR/PI3K/AKT/mTOR pathway to inhibit cell proliferation through *in vitro* experiments.

Among the active ingredients of MCLD, quercetin has various biological activities, including anti-inflammatory, antibacterial, antioxidant, and protective effects on the gastric mucosa [31]. Significantly, it has been shown to inhibit chenodeoxycholic acid (CDCA)-induced GIM by regulating the key mediator of apoptosis, caspase-3 [32], and may achieve cytoprotection through the AKT/FOXO3 pathway [33]. Kaempferol also has pharmacological activities of anti-inflammatory, antioxidant, and protective effects on damaged cells [34]. According to reports, it can protect the gastric mucosa and treat CAG by regulating the Hedgehog signaling pathway to reduce levels of IL-1β and IL-6, and it can also alleviate chronic inflammation by inhibiting the TNF-α-induced MAPK pathway [35, 36]. Isorhamnetin exerts various biological effects such as anti-inflammatory, antioxidant, antibacterial, and anticancer [37]. It may inhibit the proliferation and survival of gastric cancer cells through the EGFR/PI3K/AKT pathway [38]. Furthermore, Helicobacter pylori (HP) infection is a well-known and important cause of GIM. Ustün et al. found that isorhamnetin exhibited a significant inhibitory effect on HP [39]. Luteolin plays a certain role in antioxidant, anti-inflammatory, antibacterial, and anticancer activities, and regulation of ROS levels, inhibition of angiogenesis, and inhibition of PI3K and STAT3 are possible mechanisms of its biological activity [40]. Meanwhile, naringenin has been reported to have a protective effect on the gastric mucosa, which is closely related to its antioxidant and anti-inflammatory effects through nuclear factor kappa-B (NF-κB) and MAPK signaling pathways [41]. These results indicate that the active components of MCLD have synergistic effects, including anti-inflammatory, antibacterial, antioxidant, cytoprotection, and protective effects on the gastric mucosa, suggesting that MCLD may be a good candidate for treating or reversing GIM, further suggesting that exploring the potential mechanism of MCLD in treating GIM is of great significance.

The intestinal-specific transcription factor CDX2 can regulate intestinal differentiation of gastric cells, and abnormal expression of CDX2 in the gastric mucosa may be an early event leading to the appearance and development of GIM [42, 43]. Jin et al. used DCA to promote the proliferation of GES-1 cells and ectopic expression of CDX2, and suggested that GIM may be induced by the STAT3 signaling pathway, a transcription factor widely involved in pro-inflammatory oncogenic cell processes [11]. Lu et al. pointed out that resveratrol may inhibit CDCA-induced CDX2 and its downstream intestinal biomarker expression through the PI3K/AKT/p-FoxO4 pathway, indicating its potential to reverse GIM [25]. Here, we obtained similar results such that 150 μM DCA promoted the proliferation of GES-1 cells and increased the mRNA and protein expression of CDX2 and MUC2 related to intestinal cell differentiation, whereas MCLD significantly decreased the above trend, indicating that MCLD also has a potential reversal effect on DCA-induced GIM. Furthermore, chronic gastric inflammation is an important factor in promoting GIM and cancer progression, and IL-6, TNF-α, and IL-1β are characteristic cytokines involved in the inflammatory response [8]. Our study found that MCLD significantly reduced DCA-induced levels of the aforementioned pro-inflammatory cytokines, indicating that MCLD had the ability to inhibit chronic inflammation of the gastric mucosa. However, the mechanism that involves the inhibition of inflammatory cytokines by MCLD needs to be explored in the near future.

Chronic inflammatory stimulation of the gastric mucosa may induce abnormal cell proliferation and differentiation, leading to GIM or GC [6, 12]. Ki-67 is an antigen related to nuclear proliferation, while Cyclin-D1 is a cell cycle regulatory factor. They are signature indicators of cell proliferation and have been reported to be highly expressed in GIM [6, 44]. EGFR is a transmembrane glycoprotein with tyrosine kinase activity that can regulate cell growth, proliferation, differentiation, and survival [45]. EGFR overexpression is closely related to the pathogenesis of GC and its precancerous stage. As the gastric mucosa transitions from chronic inflammation to GC, the expression level of EGFR tends to increase, accompanied by increased proliferation of gastric mucosal cells [46], and its downstream PI3K/AKT signaling axis can be abnormally activated during this process [47]. Activated AKT (also known as protein kinase B) can further phosphorylate the mammalian target of rapamycin (mTOR) and promote cell proliferation, migration, and differentiation [48]. The EGFR/PI3K/AKT pathway is abnormally activated in the PLGC rat model (including CAG, GIM, and dysplasia), and inhibiting this pathway can effectively improve PLGC [4]. Meanwhile, a key report suggested that BA may promote GIM through the EGFR signaling pathway [9]. Here, our experimental results indicate that DCA can abnormally activate the EGFR/PI3K/AKT/mTOR pathway, promote protein expression of CDX2, MUC2, Ki-67, Cyclin-D1, and induce GIM. After treating DCA-induced GES-1 cells with MCLD, the above effects were inhibited and GIM improved. Furthermore, we combined EGFR agonists and inhibitors to treat cells and found that, as predicted by network pharmacology, MCLD may improve GIM by acting on the PI3K/AKT signaling axis through multiple pathways, one of which may be the EGFR/PI3K/AKT/mTOR pathway, which may be related to its inhibition of DCA-induced cell proliferation through this pathway.

**Figure 11:**
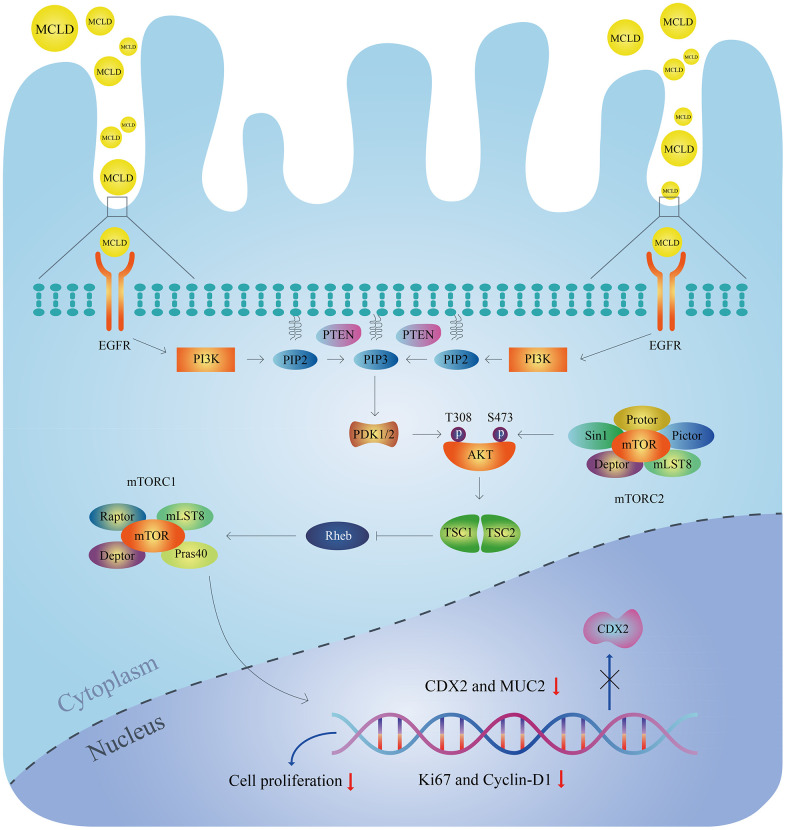
Molecular mechanisms of MCLD to improve DCA-induced GIM.

In conclusion, this study combined network pharmacology and experimental validation to reveal the molecular mechanisms of MCLD underlying its potential as an effective treatment for GIM (Figure 11). We found that MCLD can improve DCA-induced inflammation, cell proliferation, and GIM through *in vitro* cell-based experiments. It is worth noting that we verified that MCLD may attenuate cell proliferation by inhibiting the EGFR/PI3K/AKT/mTOR pathway as predicted by network pharmacology, thereby protecting gastric epithelial cells and improving GIM. MCLD has shown potential positive effects in the treatment of BA-induced GIM, which may be related to alleviating inflammation and inhibiting cell proliferation. However, there are some limitations to this study: (i) the predicted active components of MCLD may not be consistent with the actual drug components metabolized in the body; (ii) the potential mechanisms of action of the active components of MCLD affecting GIM are still unclear; (iii) the mechanisms by which MCLD improves DCA-induced inflammation remains to be clarified. Therefore, in future research, it will be necessary to evaluate the active components of MCLD *in vivo* and verify its potential mechanism of action. Furthermore, the mechanism by which MCLD improves gastric mucosal inflammation will also be the focus of future research.

## MATERIALS AND METHODS

### Drug preparation

MCLD was provided by the Affiliated Hospital of Changchun University of Traditional Chinese Medicine and was prepared from Radix bupleuri, Radix Paeoniae Alba, Codonopsis pilosula, Poria cocos, Atractylodes macrocephala, Pinellia ternata, tangerine peel, Coix seed, Fructus aurantii, Rhizoma cyperi, Radix notoginseng, Rhizoma zedoariae, and Herba hedyotis diffusa at a ratio of 10: 12: 15: 15: 15: 9: 10: 20: 10: 10: 3: 9: 20. After various Chinese herbal medicines were combined, an aqueous extract of MCLD was obtained using the Chinese herbal decoction method. The decoction was then filtered and centrifuged, and a rotary evaporator and a freeze-dryer were used successively to obtain the freeze-dried powder sample. Before the experiment, the freeze-dried powder was diluted in the culture medium according to the proportion required for the experiment. DCA (Merck Millipore, USA) is a cytotoxic bile acid that induces GIM. NSC228155 (MedChemExpress, USA) is an EGFR agonist. Erlotinib (MedChemExpress, USA) is an EGFR inhibitor. All treatments were dissolved in dimethylsulfoxide (DMSO) for the *in vitro* studies.

### Screening of active compounds and related target proteins from MCLD

We first accurately weighed MCLD and added 500 μL extraction solution (methanol:water=4:1, internal standard concentration of 10 μg/mL). After ultrasonic treatment in an ice water bath, the solution was centrifuged for 15 minutes at a speed of 12,000 rpm and a temperature of 4° C. The supernatant was then filtered using a 0.22 μm filter membrane to prepare each test sample separately. Next, the components of MCLD were identified using UPLC-Q-TOF-MS/MS [4]. Based on two important parameters of ADME (OB and DL), the components were screened using the database of Traditional Chinese Medicine Systems Pharmacology (TCMSP). Compounds that met the criteria of OB ≥30% and DL ≥0.18 criteria were considered active candidate compounds in MCLD [49]. Subsequently, the PubChem database (https://pubchem.ncbi.nlm.nih.gov/) [50] and the Swiss Target Prediction database (http://www.swisstargetprediction.ch/) [51] were combined to predict relevant targets corresponding to active compounds, and the UniProt database (http://www.uniprot.org/) [52] was used to standardize the target genes, after which duplicate targets were deleted.

### Identification of GIM-related target proteins

The GeneCards database (https://www.genecards.org) [53] and the OMIM database (https://www.omim.org/ and https://www.omim.org/search/advanced/geneMap) [54] were used to identify GIM-related target proteins. The targets obtained were based on GeneCards database scores greater than the median as the filter condition. We then merged and sorted the GIM-related target information obtained from the above databases and deleted duplicate targets.

### Construction of the protein-protein interaction network and screening of the core targets

We used Venny online software (https://bioinfogp.cnb.csic.es/tools/venny/index.html) to obtain potential targets for MCLD that act on GIM and entered these targets into the STRING database tool (https://string-db.org/) [55] to construct the PPI. Here, the species was limited to *Homo sapiens,* and the confidence score was set at greater than 0.400. Next, we saved the PPI results in the TSV file format and imported them into Cytoscape V3.7.2 [56] for the construction and analysis of the PPI network. Finally, the CytoNCA plug-in was used for the topological analysis of relevant parameters, and targets with values greater than the median of degree centrality, betweenness centrality, and closeness centrality values were taken as core targets.

### Bioinformatics analysis based on target proteins

Metascape (http://metascape.org/gp/index.html) [57] is a full-featured gene function annotation and analysis platform that updates data monthly to improve their reliability. The platform was used for enrichment analyses of the GO and KEGG pathways. The study set species as “Homo sapiens” and *P* <0.01 as screening conditions for enrichment analysis and we used the online bioinformatics tool (http://bioinformatics.com.cn/) to visualize the enrichment results.

### Molecular docking between main active ingredients and core targets

To clarify the interaction between the main active ingredients and the pathway target proteins predicted by network pharmacology, we used AutoDock Vina software (http://vina.scripps.edu/) [58] to perform molecular coupling analyses using the main active ingredients and the potential pathway target proteins. First, we obtained the three-dimensional crystal structure of the target proteins from the Protein Data Bank (PDB) database (https://www.rcsb.org/) and performed pretreatment evaluations, including water removal, hydrogenation, charge calculation, and storage conditions (PDB format). The structures of the compounds obtained from the PubChem database were optimized and saved in pdb format. Finally, AutoDock Vina was used to calculate the docking activity of target and compound structures. The lower the binding energy, the more stable the binding conformation of the receptor and the ligand [28].

### Cell culture

GES-1 human gastric mucosal epithelial cells were purchased from Fenghui Biotechnology Co., Ltd. (Hunan, China) and identified using short tandem repeat DNA analysis. GES-1 cells were cultured in Dulbecco's Modified Eagle Medium (Thermo Fisher Scientific, USA) containing 10% fetal bovine serum (FBS) (Thermo Fisher Scientific, USA) and supplemented with 100 U/mL penicillin and 100 μg/mL streptomycin. Subsequently, they were incubated in a humidified incubator at 37° C and 5% CO_2_, and cells with logarithmic growth period and good condition were taken for subsequent experiments. To explore the effects of MCLD and its mechanism of action, GES-1 cells from the control group were cultured in DMEM medium for 24 h. Meanwhile, cells from the experimental group were cultured with different combinations of DCA (50, 100, and 150 μM), MCLD (550, 100, and 150 μg/mL), erlotinib (2 μM), and NSC228155 (100 μM) for 24 h.

### Determination of cell viability

GES-1 cells were seeded in 96-well plates at a density of 1×10^4^ cells/well. After the cells adhered to the plates, we added different concentrations of DCA (100–800 μM) and MCLD (25–600 μg/mL) to incubate the cells at different times and set three wells for each concentration. Next, after removing and discarding the culture medium, we added a 10 μL MTT solution (Solarbio, Beijing) for 4 h to obtain a precipitate. The resulting solution in each well was discarded and 150 μL DMSO was added. Subsequently, the plates were placed on a table shaker at room temperature + 5° C–60° C for 10 min before measuring the absorbance of each well at 490 nm using a microplate reader (BioTek, USA).

### Enzyme-linked immunosorbent assays

GES-1 cells were inoculated in a 6-well plate at a density of 1×10^6^ cells/well for 24 h. After the cells adhered to the plate, GES-1 cells were treated with 150 μM DCA for 24 h in the presence or absence of MCLD (50, 100, and 150 μg/mL). Next, we collected the cell supernatant, which was centrifuged before performing the ELISA with strict adherence to the manufacturer’s instructions to determine the contents of IL-6, IL1β, and TNF-α in the cell supernatant.

### Total RNA extraction and RT-qPCR

TRIZOL^®^ reagent (TIANGEN, Beijing, China) was used to extract total RNA from each treatment group of GES-1 cells. A reverse transcription reaction solution was then prepared to reverse transcribe the RNA into cDNA. RevertAid reverse transcriptase was purchased from Thermo Fisher Scientificm USA. Next, SYBR Green Master Mix (Thermo Scientific, USA) and a Real-Time PCR Detection system were used for qPCR. Finally, using GAPDH as the internal reference gene, we used the 2^-∆∆Ct^ method to calculate the relative mRNA expression of each gene to be tested [28]. The PCR primers were as follows: CDX2, 5’-GGTTTCAGAACCGCAGAGCA-3’-(Forward), and 5’-GAAGACACCGGACTCAAGGG-3’-(Reverse); MUC2, 5’-TCAAAAGCAGCGTGTTCAGC-3’-(Forward), and 5’-AGCAGAAGCACTCACAGTCC-3’-(Reverse); EGFR, 5’-CAGATCGCAAAGGGCATGAA-3’-(Forward), and 5’-TTGCCTCCTTCTGCATGGTA-3’-(Reverse); PI3K, 5’-TTATAAACGAGAACGTGTG-3’-(Forward), and 5’-AATAGCTAGATAAGCC-3’-(Reverse); AKT, 5’-CAGCATCGCTTCTTTGCCGGTA-3’-(Forward), and 5’-CCTGGTGTCAGTCTCCGACGTGA-3’-(Reverse); mTOR, 5’-GTGGTGGCAGATGTGCTTAG-3’-(Forward), and 5’-TTCAGAGCCACAAACAAGGC-3’-(Reverse); GAPDH, 5’-CACCATCTTCCAGGAGCGAGA-3’-(Forward), and 5’-CATGACGAACATGGGGGCAT-3’-(Reverse).

### Western blotting analysis

Proteins were extracted from treated GES-1 cells using a radioimmunoprecipitation assay (RIPA) buffer (Beyotime, Shanghai, China) containing protease and phosphatase inhibitors. A BCA protein concentration determination kit (Beyotime, Shanghai, China) was then used to determine the protein concentration in each group. Proteins were separated by SDS-PAGE and transferred to a methanol-pre-activated PVDF membrane (Merck Millipore, USA). Finally, the PVDF membrane was completely immersed in 5% milk-PBST at room temperature for 60 min for blocking, and then the membrane and the first antibody were incubated overnight at 4° C. A secondary antibody was added to the membrane after washing for 1 h the next day. After washing again, the enhanced chemiluminescence (ECL) method was used to image the chemiluminescence (Shanghai Qinxiang Scientific Instrument Co., Ltd., China) and densitometric analysis of the protein bands was performed using ImageLab software. The primary antibodies used were anti-EGFR (# 4267, CST), anti-p-EGFR (# 3777 CST), anti-PI3K (# 60225-1-Ig, Protentech), anti-AKT (# 4691, CST), anti-p-AKT (# 4060, CST), anti-mTOR (# 2983, CST), anti-p-mTOR (# 5536, CST), anti-CDX2 (# ab76541, Abcam), anti-MUC2 (# ab272692, Abcam), anti-Ki67 (# ab92742, Abcam), anti-Cyclin-D1 (# ab134175, Abcam), and anti-GAPDH (# 60004-1-Ig, Protentech).

### Statistical analysis

Statistical analysis of all data was performed using the SPSS software (v.19.0, IBM Corp., Armonk, NY, USA). The experimental data were compared using one-way analysis of variance and expressed as mean±standard deviation (SD). Differences were considered statistically significant at *P*-values < 0.05.

## Supplementary Material

Supplementary Table 1

Supplementary Table 2

Supplementary Table 3

Supplementary Table 4
