# Supplementary material for: Modified Chaishao Liujunzi Decoction inhibits bile acid-induced gastric intestinal metaplasia: from network prediction to experimental verification
Source: Aging (Albany NY). 2023 Dec 10;15(23):13998–4018. doi: 10.18632/aging.205285 (PMC10756100; doi:10.18632/aging.205285)
Supplement: Supplementary Table 1 [file aging-15-205285-s001.docx]

**Supplementary Table 1.** Chemical profiling in the water extract of MCLD identified by UPLC-Q-TOF-MS/MS.

| **No.** | **Peak appearance time (min)** | **Ion species** | **Molecular**  **formula** | **Measured value (m/s)** | **Deviation ΔPPM** | **MS^n^**  **fragments^b^** | **Identified compounds** | **CompositeScore** |
| --- | --- | --- | --- | --- | --- | --- | --- | --- |
| 1 | 0.63 | [M+H]+ | C_5_H_5_N_5_ | 136.0619 | 0.9 | 136.061225;91.054092;119.048414;137.046359;94.039476 | Adenine | 0.97 |
| 2 | 1.02 | [M-H]- | C_7_H_6_O_5_ | 169.0140 | 0.2 | 125.024683;169.014758;97.029198;69.034417;81.034483 | Gallic acid | 0.93 |
| 3 | 1.13 | [M+H]+ | C_9_H_18_O | 143.1431 | 0.8 | 143.033258;144.101799;69.069808;125.022429;57.069837 | Pelargonaldehyde | 1.00 |
| 4 | 2.93 | [M-H]- | C_7_H_7_NO_2_ | 136.0403 | 2.3 | 136.040948;93.034565;137.024434;94.029727;122.894084 | ANTHRANILATE | 0.98 |
| 5 | 3.20 | [M-H]- | C_8_H_8_O_5_ | 183.0295 | 2.5 | 183.028541;168.006913;124.016377;140.011157;92.687602 | Methylgallate | 0.96 |
| 6 | 3.77 | [M+H]+ | C_9_H_8_O | 133.0648 | 1.9 | 133.065101;105.069528;91.05409;79.05392;103.053901 | Cinnamaldehyde | 0.96 |
| 7 | 3.87 | [M+H]+ | C_16_H_18_O_9_ | 355.1023 | 0.7 | 193.049693;133.028528;178.026666;103.075733;355.126504 | Scopolin | 0.98 |
| 8 | 4.96 | [M+H]+ | C_15_H_10_O_5_ | 271.0600 | 0.1 | 271.060297;151.038238;92.663933;178.885877;85.922683 | Aloe-emodin | 0.96 |
| 9 | 5.65 | [M+H]+ | C_8_H_10_ | 107.0854 | 4.1 | 107.085831;91.054095;105.069458;109.064654;79.053998 | m-Xylene | 1.00 |
| 10 | 5.68 | [M-H]- | C_11_H_12_O_5_ | 223.0612 | 0.9 | 208.037965;223.062449;193.013858;164.046922;149.024471 | Sinapic acid | 0.99 |
| 11 | 5.80 | [M+H]+ | C_15_H_10_O_7_ | 303.0495 | 1.8 | 303.047596;153.018402;92.667308;229.048697;257.043031 | Quercetin | 0.98 |
| 12 | 5.90 | [M+NH4]+ | C_23_H_28_O_11_ | 498.1964 | 0.9 | 179.069678;151.075123;133.064917;85.027785;135.080541 | Paeoniflorin | 0.96 |
| 13 | 6.11 | [M+H]+ | C_15_H_10_O_6_ | 287.0547 | 0.9 | 287.053032;153.018679;229.575605;67.01783;92.668137 | Kaempferol | 0.98 |
| 14 | 6.28 | [M+H]+ | C_15_H_10_O_7_ | 303.0494 | 1.9 | 303.047581;229.048473;153.01879;257.042673;92.667306 | Morin | 0.99 |
| 15 | 6.58 | [M-H]- | C_7_H_6_O_3_ | 137.0243 | 2.1 | 93.034643;137.024424;51.593029;78.805636;67.956551 | 4-Hydroxybenzoic acid | 0.96 |
| 16 | 6.62 | [M-H]- | C_16_H_12_O_5_ | 283.0608 | 0.5 | 268.035846;283.058831;211.038256;92.683393;239.033823 | Wogonin | 0.99 |
| 17 | 6.72 | [M+H]+ | C_16_H_12_O_6_ | 301.0702 | 0.7 | 301.070838;286.045533;105.032936;283.060085;258.050499 | Kaempferide | 0.96 |
| 18 | 6.91 | [M-H]- | C_16_H_14_O_6_ | 301.0707 | 1.1 | 301.073178;164.011179;286.049855;151.00374;242.059038 | Hesperetin | 0.97 |
| 19 | 7.03 | [M-H]- | C_15_H_8_O_6_ | 283.0248 | 0.5 | 283.022686;239.033838;167.050358;210.032961;179.012373 | Rheic acid | 0.99 |
| 20 | 7.22 | [M-H]- | C_15_H_10_O_6_ | 285.0403 | 1.1 | 197.061504;121.028969;285.171082;241.050616;141.091834 | Luteolin | 1.00 |
| 21 | 7.61 | [M+H]+ | C_15_H_18_O_2_ | 231.1379 | 0.3 | 231.137475;185.133229;145.101315;119.085389;203.142126 | Lindenenol | 0.95 |
| 22 | 7.61 | [M-H]- | C_15_H_8_O_7_ | 299.0196 | 1.5 | 299.018065;271.023592;199.038933;227.036034;243.029381 | Demethylwedelolactone | 0.97 |
| 23 | 7.82 | [M+H]+ | C_28_H_34_O_14_ | 595.2025 | 0.8 | 287.089614;85.027747;71.048914;153.018444;195.028662 | Didymin | 0.97 |
| 24 | 7.90 | [M+H]+ | C_15_H_12_O_4_ | 257.0807 | 1.2 | 257.08207;153.018767;171.028561;131.048693;92.663925 | Pinocembrin | 0.92 |
| 25 | 8.37 | [M+H]+ | C_15_H_16_O_4_ | 261.1122 | 0.7 | 189.055063;131.04842;261.113173;243.100165;92.66816 | Isomeranzin | 0.93 |
| 26 | 8.41 | [M+H]+ | C_15_H_12_O_5_ | 273.0754 | 1.4 | 273.07374;153.018737;147.043687;119.048512;171.027704 | Naringenin | 0.98 |
| 27 | 8.51 | [M-H]- | C_15_H_10_O_5_ | 269.0456 | 1.4 | 269.045913;251.036979;92.686779;223.040336;239.033827 | Aloeemodin | 1.00 |
| 28 | 8.76 | [M+H]+ | C_15_H_20_O_2_ | 233.1533 | 1.2 | 233.153517;215.144123;187.146791;107.084802;173.133025 | Isoalantolactone | 0.96 |
| 29 | 8.78 | [M-H]- | C_16_H_12_O_7_ | 315.0506 | 1.4 | 315.052078;300.025996;157.087172;151.003026;197.1168 | Isorhamnetin | 1.00 |
| 30 | 8.99 | [M-H]- | C_15_H_8_O_5_ | 267.0302 | 0.6 | 223.040218;267.159901;195.044253;59.013621;249.1508 | Coumestrol | 1.00 |
| 31 | 9.20 | [M-H]- | C_10_H_12_O_4_ | 195.0660 | 0.0 | 160.84269;195.81091;195.064932;180.043224;130.993137 | Methyl beta-orcinolcarboxylate | 1.00 |
| 32 | 9.26 | [M+H]+ | C_11_H_6_O_3_ | 187.0388 | 1.1 | 187.037608;131.048346;159.043756;145.101713;55.934324 | Psoralen | 1.00 |
| 33 | 9.41 | [M+FA]- | C_26_H_30_O_8_ | 515.1920 | 0.1 | 469.184867;515.303515;326.122181;275.920992;238.725711 | Limonin | 1.00 |
| 34 | 10.32 | [M+Na]+ | C_15_H_22_O_2_ | 257.1512 | 0.7 | 257.152368;258.34564;95.055282;92.696911;65.860678 | Curcumenol | 1.00 |
| 35 | 10.46 | [M+H]+ | C_17_H_24_O_3_ | 277.1795 | 1.7 | 276.281534;277.281823;137.060164;88.075231;258.271479 | shogaol | 1.00 |
| 36 | 10.49 | [M+H]+ | C_15_H_10_O_3_ | 239.0699 | 0.6 | 239.071545;211.075077;165.068754;105.033737;183.079103 | 3-Hydroxyflavone | 0.91 |
| 37 | 10.82 | [M-H]- | C_16_H_14_O_4_ | 269.0820 | 0.2 | 269.083049;254.058235;228.988327;210.067432;248.997276 | isoimperatorin | 0.93 |
| 38 | 10.88 | [M+H]+ | C_8_H_10_ | 107.0853 | 2.7 | 91.054017;107.084798;105.06958;79.053912;109.064628 | o-Xylene | 0.98 |
| 39 | 10.90 | [M+H]+ | C_15_H_20_O_3_ | 249.1484 | 1.6 | 231.13728;163.075449;69.069732;175.074284;189.092205 | Atractylenolide III | 0.98 |
| 40 | 10.95 | [M+FA]- | C_42_H_72_O_13_ | 829.4958 | 0.9 | 829.49596;829.518617;136.282119;63.852376 | Ginsenoside F2 | 0.99 |
| 41 | 11.34 | [M-H]- | C_16_H_12_O_5_ | 283.0609 | 0.5 | 283.062987;268.039538;240.042543;92.685921;242.986281 | Inermin | 0.96 |
| 42 | 12.14 | [M+H]+ | C_14_H_12_O_4_ | 245.0809 | 0.5 | 245.078603;175.03898;217.084631;86.457775;105.372503 | Oxyresveratrol | 0.95 |
| 43 | 12.87 | [M-H]- | C_14_H_22_O_2_ | 221.1544 | 1.9 | 221.153823;222.158394;205.121084;154.992964;204.116258 | Isokobusone | 1.00 |
| 44 | 12.88 | [M+H]+ | C_15_H_18_O_2_ | 231.1380 | 0.0 | 231.137367;185.132891;92.664742;157.100236;213.129012 | Atractylenolide I | 0.97 |
| 45 | 19.43 | [M+NH4]+ | C_9_H_10_O_4_ | 200.0929 | 0.7 | 200.093159;160.073604;159.066413;133.063458;118.039659 | 4-O-Methylphloracetophenone | 1.00 |
| 46 | 26.87 | [M+H]+ | C_7_H_10_N_2_ | 123.0917 | 2.8 | 82.052582;124.086384;123.092099;72.936962;81.044746 | Trimethylpyrazine | 1.00 |
