# Supplementary material for: Modified Chaishao Liujunzi Decoction inhibits bile acid-induced gastric intestinal metaplasia: from network prediction to experimental verification
Source: Aging (Albany NY). 2023 Dec 10;15(23):13998–4018. doi: 10.18632/aging.205285 (PMC10756100; doi:10.18632/aging.205285)
Supplement: Supplementary Table 2 [file aging-15-205285-s002.docx]

**Supplementary Table 2.** 141 MCLD active compounds were screened in TCMSP database based on ADME process.

| **Medicine** | **Mol ID** | **Compound** | **OB** | **DL** |
| --- | --- | --- | --- | --- |
| Chaihu | MOL001645 | [Linoleyl acetate](https://old.tcmsp-e.com/molecule.php?qn=1645) | 42.1 | 0.2 |
| Chaihu | MOL002776 | [Baicalin](https://old.tcmsp-e.com/molecule.php?qn=2776) | 40.12 | 0.75 |
| Chaihu | MOL000449 | [Stigmasterol](https://old.tcmsp-e.com/molecule.php?qn=449) | 43.83 | 0.76 |
| Chaihu | MOL000354 | [isorhamnetin](https://old.tcmsp-e.com/molecule.php?qn=354) | 49.6 | 0.31 |
| Chaihu | MOL000422 | [kaempferol](https://old.tcmsp-e.com/molecule.php?qn=422) | 41.88 | 0.24 |
| Chaihu | MOL004598 | [3,5,6,7-tetramethoxy-2-(3,4,5-trimethoxyphenyl)chromone](https://old.tcmsp-e.com/molecule.php?qn=4598) | 31.97 | 0.59 |
| Chaihu | MOL004609 | [Areapillin](https://old.tcmsp-e.com/molecule.php?qn=4609) | 48.96 | 0.41 |
| Chaihu | MOL013187 | [Cubebin](https://old.tcmsp-e.com/molecule.php?qn=13187) | 57.13 | 0.64 |
| Chaihu | MOL004624 | [Longikaurin A](https://old.tcmsp-e.com/molecule.php?qn=4624) | 47.72 | 0.53 |
| Chaihu | MOL004628 | [Octalupine](https://old.tcmsp-e.com/molecule.php?qn=4628) | 47.82 | 0.28 |
| Chaihu | MOL004644 | [Sainfuran](https://old.tcmsp-e.com/molecule.php?qn=4644) | 79.91 | 0.23 |
| Chaihu | MOL004648 | [Troxerutin](https://old.tcmsp-e.com/molecule.php?qn=4648) | 31.6 | 0.28 |
| Chaihu | MOL004653 | [(+)-Anomalin](https://old.tcmsp-e.com/molecule.php?qn=4653) | 46.06 | 0.66 |
| Chaihu | MOL004702 | [saikosaponin c_qt](https://old.tcmsp-e.com/molecule.php?qn=4702) | 30.5 | 0.63 |
| Chaihu | MOL004718 | [α-spinasterol](https://old.tcmsp-e.com/molecule.php?qn=4718) | 42.98 | 0.76 |
| Chaihu | MOL000490 | [petunidin](https://old.tcmsp-e.com/molecule.php?qn=490) | 30.05 | 0.31 |
| Chaihu | MOL000098 | [quercetin](https://old.tcmsp-e.com/molecule.php?qn=98) | 46.43 | 0.28 |
| Baishao | MOL001910 | [11alpha,12alpha-epoxy-3beta-23-dihydroxy-30-norolean-20-en-28,12beta-olide](https://old.tcmsp-e.com/molecule.php?qn=1910) | 64.77 | 0.38 |
| Baishao | MOL001918 | [paeoniflorgenone](https://old.tcmsp-e.com/molecule.php?qn=1918) | 87.59 | 0.37 |
| Baishao | MOL001919 | [(3S,5R,8R,9R,10S,14S)-3,17-dihydroxy-4,4,8,10,14-pentamethyl-2,3,5,6,7,9-hexahydro-1H-cyclopenta[a]phenanthrene-15,16-dione](https://old.tcmsp-e.com/molecule.php?qn=1919) | 43.56 | 0.53 |
| Baishao | MOL001921 | [Lactiflorin](https://old.tcmsp-e.com/molecule.php?qn=1921) | 49.12 | 0.8 |
| Baishao | MOL001924 | [paeoniflorin](https://old.tcmsp-e.com/molecule.php?qn=1924) | 53.87 | 0.79 |
| Baishao | MOL001925 | [paeoniflorin_qt](https://old.tcmsp-e.com/molecule.php?qn=1925) | 68.18 | 0.4 |
| Baishao | MOL001928 | [albiflorin_qt](https://old.tcmsp-e.com/molecule.php?qn=1928) | 66.64 | 0.33 |
| Baishao | MOL001930 | [benzoyl paeoniflorin](https://old.tcmsp-e.com/molecule.php?qn=1930) | 31.27 | 0.75 |
| Baishao | MOL000211 | [Mairin](https://old.tcmsp-e.com/molecule.php?qn=211) | 55.38 | 0.78 |
| Baishao | MOL000358 | [beta-sitosterol](https://old.tcmsp-e.com/molecule.php?qn=358) | 36.91 | 0.75 |
| Baishao | MOL000359 | [sitosterol](https://old.tcmsp-e.com/molecule.php?qn=359) | 36.91 | 0.75 |
| Baishao | MOL000422 | [kaempferol](https://old.tcmsp-e.com/molecule.php?qn=422) | 41.88 | 0.24 |
| Baishao | MOL000492 | [(+)-catechin](https://old.tcmsp-e.com/molecule.php?qn=492) | 54.83 | 0.24 |
| Dangshen | MOL001006 | poriferasta-7,22E-dien-3beta-ol | 42.98 | 0.76 |
| Dangshen | MOL002140 | Perlolyrine | 65.95 | 0.27 |
| Dangshen | MOL002879 | Diop | 43.59 | 0.39 |
| Dangshen | MOL003036 | ZINC03978781 | 43.83 | 0.76 |
| Dangshen | MOL000449 | Stigmasterol | 43.83 | 0.76 |
| Dangshen | MOL003896 | 7-Methoxy-2-methyl isoflavone | 42.56 | 0.2 |
| Dangshen | MOL004355 | Spinasterol | 42.98 | 0.76 |
| Dangshen | MOL004492 | Chrysanthemaxanthin | 38.72 | 0.58 |
| Dangshen | MOL005321 | Frutinone A | 65.9 | 0.34 |
| Dangshen | MOL000006 | luteolin | 36.16 | 0.25 |
| Dangshen | MOL006554 | Taraxerol | 38.4 | 0.77 |
| Dangshen | MOL006774 | stigmast-7-enol | 37.42 | 0.75 |
| Dangshen | MOL007059 | 3-beta-Hydroxymethyllenetanshiquinone | 32.16 | 0.41 |
| Dangshen | MOL007514 | methyl icosa-11,14-dienoate | 39.67 | 0.23 |
| Dangshen | MOL008391 | 5alpha-Stigmastan-3,6-dione | 33.12 | 0.79 |
| Dangshen | MOL008393 | 7-(beta-Xylosyl)cephalomannine_qt | 38.33 | 0.29 |
| Dangshen | MOL008397 | Daturilin | 50.37 | 0.77 |
| Dangshen | MOL008400 | glycitein | 50.48 | 0.24 |
| Dangshen | MOL008406 | Spinoside A | 39.97 | 0.4 |
| Dangshen | MOL008407 | (8S,9S,10R,13R,14S,17R)-17-[(E,2R,5S)-5-ethyl-6-methylhept-3-en-2-yl]-10,13-dimethyl-1,2,4,7,8,9,11,12,14,15,16,17-dodecahydrocyclopenta[a]phenanthren-3-one | 45.4 | 0.76 |
| Dangshen | MOL008411 | 11-Hydroxyrankinidine | 40 | 0.66 |
| Fuling | MOL000273 | [(2R)-2-[(3S,5R,10S,13R,14R,16R,17R)-3,16-dihydroxy-4,4,10,13,14-pentamethyl-2,3,5,6,12,15,16,17-octahydro-1H-cyclopenta[a]phenanthren-17-yl]-6-methylhept-5-enoic acid](https://old.tcmsp-e.com/molecule.php?qn=273) | 30.93 | 0.81 |
| Fuling | MOL000275 | [trametenolic acid](https://old.tcmsp-e.com/molecule.php?qn=275) | 38.71 | 0.8 |
| Fuling | MOL000276 | [7,9(11)-dehydropachymic acid](https://old.tcmsp-e.com/molecule.php?qn=276) | 35.11 | 0.81 |
| Fuling | MOL000279 | [Cerevisterol](https://old.tcmsp-e.com/molecule.php?qn=279) | 37.96 | 0.77 |
| Fuling | MOL000280 | [(2R)-2-[(3S,5R,10S,13R,14R,16R,17R)-3,16-dihydroxy-4,4,10,13,14-pentamethyl-2,3,5,6,12,15,16,17-octahydro-1H-cyclopenta[a]phenanthren-17-yl]-5-isopropyl-hex-5-enoic acid](https://old.tcmsp-e.com/molecule.php?qn=280) | 31.07 | 0.82 |
| Fuling | MOL000282 | [ergosta-7,22E-dien-3beta-ol](https://old.tcmsp-e.com/molecule.php?qn=282) | 43.51 | 0.72 |
| Fuling | MOL000283 | [Ergosterol peroxide](https://old.tcmsp-e.com/molecule.php?qn=283) | 40.36 | 0.81 |
| Fuling | MOL000285 | [(2R)-2-[(5R,10S,13R,14R,16R,17R)-16-hydroxy-3-keto-4,4,10,13,14-pentamethyl-1,2,5,6,12,15,16,17-octahydrocyclopenta[a]phenanthren-17-yl]-5-isopropyl-hex-5-enoic acid](https://old.tcmsp-e.com/molecule.php?qn=285) | 38.26 | 0.82 |
| Fuling | MOL000287 | [3beta-Hydroxy-24-methylene-8-lanostene-21-oic acid](https://old.tcmsp-e.com/molecule.php?qn=287) | 38.7 | 0.81 |
| Fuling | MOL000289 | [pachymic acid](https://old.tcmsp-e.com/molecule.php?qn=289) | 33.63 | 0.81 |
| Fuling | MOL000290 | [Poricoic acid A](https://old.tcmsp-e.com/molecule.php?qn=290) | 30.61 | 0.76 |
| Fuling | MOL000291 | [Poricoic acid B](https://old.tcmsp-e.com/molecule.php?qn=291) | 30.52 | 0.75 |
| Fuling | MOL000292 | [poricoic acid C](https://old.tcmsp-e.com/molecule.php?qn=292) | 38.15 | 0.75 |
| Fuling | MOL000296 | [hederagenin](https://old.tcmsp-e.com/molecule.php?qn=296) | 36.91 | 0.75 |
| Fuling | MOL000300 | [dehydroeburicoic acid](https://old.tcmsp-e.com/molecule.php?qn=300) | 44.17 | 0.83 |
| Baizhu | MOL000020 | [12-senecioyl-2E,8E,10E-atractylentriol](https://old.tcmsp-e.com/molecule.php?qn=20) | 62.4 | 0.22 |
| Baizhu | MOL000021 | [14-acetyl-12-senecioyl-2E,8E,10E-atractylentriol](https://old.tcmsp-e.com/molecule.php?qn=21) | 60.31 | 0.31 |
| Baizhu | MOL000022 | [14-acetyl-12-senecioyl-2E,8Z,10E-atractylentriol](https://old.tcmsp-e.com/molecule.php?qn=22) | 63.37 | 0.3 |
| Baizhu | MOL000028 | [α-Amyrin](https://old.tcmsp-e.com/molecule.php?qn=28) | 39.51 | 0.76 |
| Baizhu | MOL000033 | [(3S,8S,9S,10R,13R,14S,17R)-10,13-dimethyl-17-[(2R,5S)-5-propan-2-yloctan-2-yl]-2,3,4,7,8,9,11,12,14,15,16,17-dodecahydro-1H-cyclopenta[a]phenanthren-3-ol](https://old.tcmsp-e.com/molecule.php?qn=33) | 36.23 | 0.78 |
| Baizhu | MOL000049 | [3β-acetoxyatractylone](https://old.tcmsp-e.com/molecule.php?qn=49) | 54.07 | 0.22 |
| Baizhu | MOL000072 | [8β-ethoxy atractylenolide Ⅲ](https://old.tcmsp-e.com/molecule.php?qn=72) | 35.95 | 0.21 |
| Banxia | MOL001755 | [24-Ethylcholest-4-en-3-one](https://old.tcmsp-e.com/molecule.php?qn=1755) | 36.08 | 0.76 |
| Banxia | MOL002670 | [Cavidine](https://old.tcmsp-e.com/molecule.php?qn=2670) | 35.64 | 0.81 |
| Banxia | MOL002714 | [baicalein](https://old.tcmsp-e.com/molecule.php?qn=2714) | 33.52 | 0.21 |
| Banxia | MOL002776 | [Baicalin](https://old.tcmsp-e.com/molecule.php?qn=2776) | 40.12 | 0.75 |
| Banxia | MOL000358 | [beta-sitosterol](https://old.tcmsp-e.com/molecule.php?qn=358) | 36.91 | 0.75 |
| Banxia | MOL000449 | [Stigmasterol](https://old.tcmsp-e.com/molecule.php?qn=449) | 43.83 | 0.76 |
| Banxia | MOL005030 | [gondoic acid](https://old.tcmsp-e.com/molecule.php?qn=5030) | 30.7 | 0.2 |
| Banxia | MOL000519 | [coniferin](https://old.tcmsp-e.com/molecule.php?qn=519) | 31.11 | 0.32 |
| Banxia | MOL006936 | [10,13-eicosadienoic](https://old.tcmsp-e.com/molecule.php?qn=6936) | 39.99 | 0.2 |
| Banxia | MOL006937 | [12,13-epoxy-9-hydroxynonadeca-7,10-dienoic acid](https://old.tcmsp-e.com/molecule.php?qn=6937) | 42.15 | 0.24 |
| Banxia | MOL006957 | [(3S,6S)-3-(benzyl)-6-(4-hydroxybenzyl)piperazine-2,5-quinone](https://old.tcmsp-e.com/molecule.php?qn=6957) | 46.89 | 0.27 |
| Banxia | MOL003578 | [Cycloartenol](https://old.tcmsp-e.com/molecule.php?qn=3578) | 38.69 | 0.78 |
| Banxia | MOL006967 | [beta-D-Ribofuranoside, xanthine-9](https://old.tcmsp-e.com/molecule.php?qn=6967) | 44.72 | 0.21 |
| Chenpi | MOL000359 | [sitosterol](https://old.tcmsp-e.com/molecule.php?qn=359) | 36.91 | 0.75 |
| Chenpi | MOL004328 | [naringenin](https://old.tcmsp-e.com/molecule.php?qn=4328) | 59.29 | 0.21 |
| Chenpi | MOL005100 | [5,7-dihydroxy-2-(3-hydroxy-4-methoxyphenyl)chroman-4-one](https://old.tcmsp-e.com/molecule.php?qn=5100) | 47.74 | 0.27 |
| Chenpi | MOL005815 | [Citromitin](https://old.tcmsp-e.com/molecule.php?qn=5815) | 86.9 | 0.51 |
| Chenpi | MOL005828 | [nobiletin](https://old.tcmsp-e.com/molecule.php?qn=5828) | 61.67 | 0.52 |
| Zhiqiao | MOL013381 | [Marmin](https://old.tcmsp-e.com/molecule.php?qn=13381) | 38.23 | 0.31 |
| Zhiqiao | MOL002341 | [Hesperetin](https://old.tcmsp-e.com/molecule.php?qn=2341) | 70.31 | 0.27 |
| Zhiqiao | MOL000358 | [beta-sitosterol](https://old.tcmsp-e.com/molecule.php?qn=358) | 36.91 | 0.75 |
| Zhiqiao | MOL004328 | [naringenin](https://old.tcmsp-e.com/molecule.php?qn=4328) | 59.29 | 0.21 |
| Zhiqiao | MOL005828 | [nobiletin](https://old.tcmsp-e.com/molecule.php?qn=5828) | 61.67 | 0.52 |
| Xiangfu | MOL003044 | Chryseriol | 35.85 | 0.27 |
| Xiangfu | MOL000354 | isorhamnetin | 49.6 | 0.31 |
| Xiangfu | MOL003542 | 8-Isopentenyl-kaempferol | 38.04 | 0.39 |
| Xiangfu | MOL000358 | beta-sitosterol | 36.91 | 0.75 |
| Xiangfu | MOL000359 | sitosterol | 36.91 | 0.75 |
| Xiangfu | MOL004027 | 1,4-Epoxy-16-hydroxyheneicos-1,3,12,14,18-pentaene | 45.1 | 0.24 |
| Xiangfu | MOL004053 | Isodalbergin | 35.45 | 0.2 |
| Xiangfu | MOL004058 | Khell | 33.19 | 0.19 |
| Xiangfu | MOL004059 | khellol glucoside | 74.96 | 0.72 |
| Xiangfu | MOL010489 | Resivit | 30.84 | 0.27 |
| Xiangfu | MOL004068 | rosenonolactone | 79.84 | 0.37 |
| Xiangfu | MOL004071 | Hyndarin | 73.94 | 0.64 |
| Xiangfu | MOL004074 | stigmasterol glucoside_qt | 43.83 | 0.76 |
| Xiangfu | MOL004077 | sugeonyl acetate | 45.08 | 0.2 |
| Xiangfu | MOL000422 | kaempferol | 41.88 | 0.24 |
| Xiangfu | MOL000449 | Stigmasterol | 43.83 | 0.76 |
| Xiangfu | MOL000006 | luteolin | 36.16 | 0.25 |
| Xiangfu | MOL000098 | quercetin | 46.43 | 0.28 |
| Sanqi | MOL001494 | [Mandenol](https://old.tcmsp-e.com/molecule.php?qn=1494) | 42 | 0.19 |
| Sanqi | MOL001792 | [DFV](https://old.tcmsp-e.com/molecule.php?qn=1792) | 32.76 | 0.18 |
| Sanqi | MOL002879 | [Diop](https://old.tcmsp-e.com/molecule.php?qn=2879) | 43.59 | 0.39 |
| Sanqi | MOL000358 | [beta-sitosterol](https://old.tcmsp-e.com/molecule.php?qn=358) | 36.91 | 0.75 |
| Sanqi | MOL000449 | [Stigmasterol](https://old.tcmsp-e.com/molecule.php?qn=449) | 43.83 | 0.76 |
| Sanqi | MOL005344 | [ginsenoside rh2](https://old.tcmsp-e.com/molecule.php?qn=5344) | 36.32 | 0.56 |
| Sanqi | MOL007475 | [ginsenoside f2](https://old.tcmsp-e.com/molecule.php?qn=7475) | 36.43 | 0.25 |
| Sanqi | MOL000098 | [quercetin](https://old.tcmsp-e.com/molecule.php?qn=98) | 46.43 | 0.28 |
| Ezhu | MOL000354 | [hederagenin](https://old.tcmsp-e.com/molecule.php?qn=296) | 36.91 | 0.75 |
| Ezhu | MOL000422 | [wenjine](https://old.tcmsp-e.com/molecule.php?qn=906) | 47.93 | 0.27 |
| Ezhu | MOL000098 | [bisdemethoxycurcumin](https://old.tcmsp-e.com/molecule.php?qn=940) | 77.38 | 0.26 |
| Baihuasheshecao | MOL001646 | [2,3-dimethoxy-6-methyanthraquinone](https://old.tcmsp-e.com/molecule.php?qn=1646) | 34.86 | 0.26 |
| Baihuasheshecao | MOL001659 | [Poriferasterol](https://old.tcmsp-e.com/molecule.php?qn=1659) | 43.83 | 0.76 |
| Baihuasheshecao | MOL001663 | [(4aS,6aR,6aS,6bR,8aR,10R,12aR,14bS)-10-hydroxy-2,2,6a,6b,9,9,12a-heptamethyl-1,3,4,5,6,6a,7,8,8a,10,11,12,13,14b-tetradecahydropicene-4a-carboxylic acid](https://old.tcmsp-e.com/molecule.php?qn=1663) | 32.03 | 0.76 |
| Baihuasheshecao | MOL001670 | [2-methoxy-3-methyl-9,10-anthraquinone](https://old.tcmsp-e.com/molecule.php?qn=1670) | 37.83 | 0.21 |
| Baihuasheshecao | MOL000449 | [Stigmasterol](https://old.tcmsp-e.com/molecule.php?qn=449) | 43.83 | 0.76 |
| Baihuasheshecao | MOL000358 | [beta-sitosterol](https://old.tcmsp-e.com/molecule.php?qn=358) | 36.91 | 0.75 |
| Baihuasheshecao | MOL000098 | [quercetin](https://old.tcmsp-e.com/molecule.php?qn=98) | 46.43 | 0.28 |
| Yiyiren | MOL001323 | [Sitosterol alpha1](https://old.tcmsp-e.com/molecule.php?qn=1323) | 43.28 | 0.78 |
| Yiyiren | MOL001494 | [Mandenol](https://old.tcmsp-e.com/molecule.php?qn=1494) | 42 | 0.19 |
| Yiyiren | MOL002372 | [(6Z,10E,14E,18E)-2,6,10,15,19,23-hexamethyltetracosa-2,6,10,14,18,22-hexaene](https://old.tcmsp-e.com/molecule.php?qn=2372) | 33.55 | 0.42 |
| Yiyiren | MOL002882 | [[(2R)-2,3-dihydroxypropyl] (Z)-octadec-9-enoate](https://old.tcmsp-e.com/molecule.php?qn=2882) | 34.13 | 0.3 |
| Yiyiren | MOL000359 | [sitosterol](https://old.tcmsp-e.com/molecule.php?qn=359) | 36.91 | 0.75 |
| Yiyiren | MOL000449 | [Stigmasterol](https://old.tcmsp-e.com/molecule.php?qn=449) | 43.83 | 0.76 |
| Yiyiren | MOL008118 | [Coixenolide](https://old.tcmsp-e.com/molecule.php?qn=8118) | 32.4 | 0.43 |
| Yiyiren | MOL008121 | [2-Monoolein](https://old.tcmsp-e.com/molecule.php?qn=8121) | 34.23 | 0.29 |
| Yiyiren | MOL000953 | [CLR](https://old.tcmsp-e.com/molecule.php?qn=953) | 37.87 | 0.68 |
